# Impact of SARS-CoV-2 variant mutations on susceptibility to monoclonal antibodies and antiviral drugs: a non-systematic review, April 2022 to October 2024

**DOI:** 10.2807/1560-7917.ES.2025.30.10.2400252

**Published:** 2025-03-13

**Authors:** Maja Vukovikj, Angeliki Melidou, Priyanka Nannapaneni, Tanja Normark, Annette Kraus, Eeva K Broberg

**Affiliations:** 1 European Centre for Disease Prevention and Control (ECDC), Stockholm, Sweden

**Keywords:** antiviral, monoclonal antibody, therapy, SARS-CoV-2, susceptibility, mutation

## Abstract

**Background:**

Monoclonal antibodies (mAbs) and antiviral drugs have emerged as additional tools for treatment of COVID-19.

**Aim:**

We aimed to review data on susceptibility of 14 SARS-CoV-2 variants to mAbs and antiviral drugs authorised in the European Union/European Economic Area (EU/EEA) countries.

**Methods:**

We constructed a literature review compiling 298 publications from four databases: PubMed, Science Direct, LitCovid and BioRxiv/MedRxiv preprint servers. We included publications on nirmatrelvir and ritonavir, remdesivir and tixagevimab and cilgavimab, regdanvimab, casirivimab and imdevimab, and sotrovimab approved by the European Medicines Agency (EMA) by 1 October 2024.

**Results:**

The mutations identified in the open reading frame (ORF)1ab, specifically nsp5:H172Y, nsp5:H172Y and Q189E, nsp5:L50F and E166V and nsp5:L50F, E166A and L167V, led to a decrease in susceptibility to nirmatrelvir and ritonavir, ranging from moderate (25-99) to high reductions (> 100). Casirivimab and imdevimab exhibited highly reduced neutralisation capacity across all Omicron sub-lineages. Sub-lineages BA.1, BA.2 and BA.5 had decreased susceptibility to regdanvimab, while sotrovimab showed decreased efficacy for BA.2, BA.4, BQ.1.1 and BA.2.86. Tixagevimab and cilgavimab exhibited highly reduced neutralisation activity against BQ.1, BQ.1.1, XBB, XBB.1.5 and BA.2.86 sub-lineages.

**Conclusions:**

The emergence of new variants, some with altered antigenic characteristics, may lead to resistance against mAbs and/or antiviral drugs and evasion of immunity induced naturally or by vaccination. This summary of mutations, combination of mutations and SARS-CoV-2 variants linked to reduced susceptibility to mAbs and antiviral drugs, should aid the selection of appropriate treatment strategies and/or phasing out therapies that have lost their effectiveness.

## Introduction

As of February 2025, > 777 million confirmed cases of COVID-19 and > 7 million deaths have been recorded [1]. Throughout the COVID-19 pandemic, various non-pharmaceutical measures, such as contact tracing, stay-at-home guidance and social distancing, were sequentially implemented to curb severe acute respiratory syndrome coronavirus 2 (SARS-CoV-2) transmission [2-4]. To mitigate the impact of the disease, vaccines, antiviral drugs and monoclonal antibodies (mAbs) were expeditiously developed. The global collaboration to develop and authorise COVID-19 vaccines within a year from the onset of the pandemic was an unprecedented achievement, considerably reducing the severity and mortality associated with the disease. At present, treatment of COVID-19 primarily involves supportive measures, including oxygen therapy for severely ill and high-risk patients and ventilation for those in critical condition [5]. Complementing these treatments, antiviral drugs and mAbs can be administered either individually or as mAb cocktails.

Antiviral drugs disrupt the life cycle of SARS-CoV-2 by hindering the attachment of the virus to host cells, preventing the proteolytic cleavage of the viral spike (S) protein and suppressing or inhibiting viral replication [6]. Among the antiviral drugs authorised for use by the European Medicines Agency (EMA) are Paxlovid (active substances: nirmatrelvir and ritonavir) and Veklury (active substance: remdesivir). Paxlovid functions as a SARS-CoV-2 protease inhibitor, with nirmatrelvir acting as the main protease (Mpro) inhibitor designed to specifically block the activity of the SARS-CoV-2 Mpro enzyme, thereby inhibiting viral replication [7]. Additionally, it contains a low dose of ritonavir, an HIV-1 protease inhibitor, and potent cytochrome P450 (CYP) 3A inhibitor, which serves as a pharmacokinetic booster for nirmatrelvir [7]. Remdesivir, on the other hand, is a nucleoside analogue that inhibits the RNA-dependent RNA polymerase (RdRp), demonstrating broad-spectrum activity against various RNA viruses, thus disrupting viral replication [8].

Monoclonal antibodies are designed to reduce the severity of COVID-19 by binding to the S protein of SARS-CoV-2 and thereby directly neutralising the virus and triggering processes like antibody-dependent cellular phagocytosis, antibody-dependent cell-mediated cytotoxicity and complement activation [9]. The European Medicines Agency has authorised mAbs for prevention or treatment of COVID-19. These mAbs, Evusheld (tixagevimab and cilgavimab), Regkirona (regdanvimab), Ronapreve (casirivimab and imdevimab) and Xevudy (sotrovimab), target either the virus itself or the S protein. However, the emergence of new SARS-CoV-2 variants with specific amino acid changes in the S protein has made the virus less susceptible to these therapies, thus raising concerns about their efficacy [10]. For instance, the reduced efficacy of Evusheld against emerging variants has limited its use. In early 2022, outbreaks linked to the Omicron (Phylogenetic Assignment of Named Global Outbreak (Pango) lineage designation B.1.1.529) lineage, including sub-lineages BA.4, BA.5, BA.2.75, XBB and XBB.1.5, occurred globally [11-13]. These variants and sub-lineages have shown decreased susceptibility to the authorised mAb therapies in vitro [14,15].

Individuals with weakened immune systems have a reduced ability to control virus replication which may result in prolonged infections and treatment and thus in a risk for development of drug resistance. Prolonged shedding of the virus may increase the risk of mutations linked to resistance and antigenic drift resulting in potential treatment failure. Therefore, patients with weakened immune systems should be tested during treatment and the specimens characterised phenotypically and genotypically. It is crucial to monitor viral levels and susceptibility to antiviral drugs in these patients to identify, evaluate and, if necessary, modify the treatment to interrupt the circulation of resistant variants. Surveillance data, alongside information on how susceptible emerging SARS-CoV-2 variants are to antiviral treatments, are crucial for decisions on the therapy [16]. However, testing of the specific variant before initiating treatment is rarely an option as resistance testing takes several days and would delay the potentially effective treatment.

Various phenotypic and genotypic laboratory techniques can be used to evaluate the ability of antibodies to neutralise a virus and its susceptibility to antiviral drugs [17]: live virus neutralisation assays, like plaque/focus reduction neutralisation tests (PRNT/FRNT), microneutralisation assay (MNA) and pseudovirus (PSV) neutralisation assays [18,19]. Replication-competent clinical SARS-CoV-2 isolates are typically used in PRNT/FRNT or TCID50 (median tissue culture infectious dose)-based assays and MNA and allow assessment against the complete antigenic content and complexity of variants. Plaque reduction neutralisation test has demonstrated higher sensitivity than other methods [20], while FRNT and MNA offer quicker results. The efficacy of antiviral drugs and resistance can be assessed by using assays to determine the inhibitory concentration (IC) which is the drug concentration needed to inhibit viral growth by 50% (IC50: half maximal inhibitory concentration). Additionally, whole genome sequencing (WGS) helps in identifying mutations in critical areas of the viral genome that might confer reduced susceptibility to antiviral drugs or mAbs.

Here, we present an overview of the currently available data on susceptibility of the different SARS-CoV-2 variants to the therapeutic mAbs and antiviral drugs authorised for use in the European Union/European Economic Area (EU/EEA) countries based on published literature. In this non-systematic literature review, we summarise the mutations, combination of mutations and SARS-CoV-2 variants linked to reduced susceptibility to mAbs and antiviral drugs, potentially aiding the selection of appropriate treatment strategies and/or phasing out of therapies that have lost efficacy.

## Methods

We constructed an in-house database by gathering data from the following sources: the Stanford University coronavirus antiviral and resistance database [21], COVID-19 Genomics UK (COG-UK) mutation explorer [22], journal publications, EMA summaries of product characteristics [23,24] and the United States Food and Drug Administration (FDA) emergency use authorisation fact sheets [11,25,26]. For our database, the inclusion criteria were limited to publications from four databases: PubMed, Science Direct, LitCovid and BioRxiv/MedRxiv preprint servers using the search terms ‘SARS-CoV-2’ combined with ‘monoclonal antibodies/antiviral drugs’. Additionally, we included only publications on antiviral drugs and mAbs approved by EMA by 1 October 2024. The data collection began in April 2022 and continued until 4 October 2024. This work is a continuation of a previous report, that covered the period April–December 2022 [27].

We regularly updated the database to incorporate new information about virus variants and research findings. Specifically, we extracted information on the types of tests used for assessing antiviral resistance, the IC50 value of the used control, fold reductions in neutralisation titre associated with different variants and mutations and the corresponding IC50 values for the quantification of the observed reduction. We collected information on mutations within the open reading frame 1ab (ORF1ab) and S genes from in vitro and in vivo studies. The database also included neutralisation data from replication-competent SARS-CoV-2 isolates and non-replication-competent pseudotyped viruses using VSV (vesicular stomatitis virus), HIV (human immunodeficiency virus) type 1, MLV (murine leukaemia virus) and a lentivirus containing a SARS-CoV-2 S protein with specific mutations.

Sequenced isolates reported to the Global Initiative on Sharing All Influenza Data (GISAID) (https://gisaid.org/) EpiCoV sequence database between 1 December 2019 and 4 October 2024 were analysed to calculate the mutation proportions. The metadata snapshot was downloaded from the GISAID EpiCoV on 5 November 2024. For each sequence, epidemiological data (like collection date and reporting country) and sequence data (e.g. Pango lineage, amino acid substitutions) were included. Mutational proportions were calculated for the aforementioned period both globally, including all available sequences, and for isolates from the EU/EEA countries only. In both instances, only human isolates were included. The proportions of single or sets of mutations were calculated based on the presence of such mutations in all available sequences for the period and regions. The lineage-specific mutational frequencies were calculated to determine the proportion of a single mutation or set of mutations within a specified Pango lineage. For these calculations, sub-lineages for each Pango lineage were included in the analysis. All calculations were performed on the data available in the GISAID snapshot.

## Results

### Overview of the literature

We screened 298 publications on the four approved mAbs and 56 publications on the two antiviral drugs (Table 1) [27]. These publications presented data on 18 distinct mutations in the S protein and 23 mutations in the ORF1ab protein. Additionally, data were gathered for 16 combinations of S protein mutations and seven mutation combinations in the ORF1ab gene linked to reduced susceptibility to monoclonal mAbs and antiviral drugs. These combinations comprised 2, 3, 15, 16 or 20 mutations found within the genome of a variant, presented in Supplementary Table 1. Of the mAb results, 194 were derived from pseudotype neutralisation assays, while the remaining 104 were from assays involving replication-competent SARS-CoV-2 isolates.

**Table 1:** Number of publications included in a review on the susceptibility monoclonal antibodies and antiviral drugs targeted against SARS-CoV-2 and approved by the European Medicines Agency, April 2022–1 November 2024 (n = 354)

Monoclonal antibodies			Antiviral drugs		
Commercial name	Active substances	Number of publications	Commercial name	Active substances	Number of publications
Evusheld	Tixagevimab and cilgavimab	84	Paxlovid	Nirmatrelvir and ritonavir	34
Regkirona	Regdanvimab	34			
Ronapreve	Casirivimab and imdevimab	72	Veklury	Remdesivir	22
Xevudy	Sotrovimab	108			

SARS-CoV-2: severe acute respiratory syndrome coronavirus 2.

### Susceptibility of monoclonal antibodies and antiviral drugs

Supplementary Table 1 provides the amino acid substitution fold changes by antiviral drug or mAb and by SARS-CoV-2 variant and the references for all the literature summarised here. Mutations E337R/L and E340K/V/A in the Delta (Pango lineage designation B.1.617.2) variant conferred ≥ 100-fold reduction in susceptibility to Xevudy (sotrovimab) [28], although the mutations were quite rare (< 0.01%). In the Beta (Pango lineage designation B.1.351) and Gamma (Pango lineage designation P.1) variants, the combination of mutations K417N, E484K and N501Y correlated with highly reduced neutralisation efficacy for Regkirona (regdanvimab) (pseudovirus: 184-fold and SARS-CoV-2: 138-fold) [23]. These mutations were more common in the Beta variant globally (80.4%) and in the EU/EEA countries (83.4%).

Another set of mutations, K417N, L452R and T478K in the Delta variant, resulted in a 183-fold decrease in susceptibility to Regkirona (regdanvimab) when comparing with wildtype SARS-CoV-2 viruses [23].

Additionally, a combination of mutations (G339D,  S371L,  S373P, S375F, K417N, N440K, G446S, S477N, T478K, E484A, Q493R, G496S, Q498R, N501Y and Y505H) found in BA.1, BA.2, BA.4 and BA.5 (number of isolates: 1,336,850; 1,712; 1; 11 on a global level, respectively) led to varying reductions in susceptibility to different mAbs. When present in the BA.1 sub-lineage, these mutations conferred ≥ 100-fold reductions in susceptibility to Ronapreve and Evusheld when using pseudotyped virus-like particles [11,25]. A total of 256,786 (48.1%) of the 533,489 isolates in the EU/EEA countries and 1,336,850 (51.5%) of the 2,594,852 global isolates carried this mutation set. On the other hand, in the sub-lineages BA.4 and BA.5, these mutations resulted in moderately reduced neutralisation efficacy (25-99) for Evusheld (tixagevimab and cilgavimab) [11] (Table 2).

**Table 2:** Mutations and their combinations in the spike protein of SARS-CoV-2 and reduction in susceptibility to monoclonal antibodies in a review on the susceptibility of monoclonal antibodies and antiviral drugs and targeted against SARS-CoV-2, April 2022–November 2024 (n = 34)^a^

Mutations in the spike protein	Variant or sub-lineage	Virus type	Reduction in susceptibility				Proportion^b^	
			Ronapreve (casirivimab and imdevimab)	Xevudy (sotrovimab)	Evusheld (tixagevimab and cilgavimab)	Regkirona (regdanvimab)	Global	EU/EEA
N501Y	Alpha	Pseudovirus	< 2	No data	< 5	No data	97.33	98.29
		Virus isolate	No data	No data	1.4	No data		
N501Y and P681H	Alpha	Pseudovirus	No data			< 5	96.58	97.25
		Virus isolate	No data			< 5		
K417N, E484K and N501Y	Beta	Pseudovirus	< 2	No data	< 5	184	80.4	83.36
		Virus isolate	No data	No data	< 5	19.7		
P337H	Delta	Pseudovirus	No data	5	No data		0.00	0.00
P337L	Delta	Pseudovirus	No data	192	No data		0.01	0.01
P337R	Delta	Pseudovirus	No data	192	No data		0.00	0.00
P337T	Delta	Pseudovirus	No data	11	No data		0.00	0.00
E340A	Delta	Pseudovirus	No data	100	No data		0.00	0.00
E340G	Delta	Pseudovirus	No data	18	No data		0.00	0.00
E340K	Delta	Pseudovirus	No data	297	No data		0.01	0.01
E340Q	Delta	Pseudovirus	No data	50	No data		0.00	0.00
E340V	Delta	Pseudovirus	No data	200	No data		0.00	0.00
K417N, L452R and T478K	Delta	Pseudovirus	No data			27.7	0.18	0.04
		Virus isolate	No data			183		
L452R and T478K	Delta	Pseudovirus	< 2	No data	< 5	No data	95.98	96.85
		Virus isolate	No data	No data	< 5	No data		
K417T, E484K and N501Y	Gamma	Pseudovirus	< 2	< 5	< 5	61.4	90.22	92.09
		Virus isolate	No data	No data	< 5	137.9		
E484A	BA.1	Pseudovirus	4.3	0.8	No data		88.35	84.3
G339D	BA.1	Pseudovirus	1.8	2	No data		93.15	91.14
G446S	BA.1	Pseudovirus	3.3	No data			70.5	78.65
K417N	BA.1	Pseudovirus	2.5	No data			66.34	71.97
N440K	BA.1	Pseudovirus	2.2	1.7	No data		69.19	76.62
S373P	BA.1	Pseudovirus	3	4.9	No data		86.35	83.33
S375F	BA.1	Pseudovirus	0.8	1.4	No data		86.28	83.99
Y505H	BA.1	Pseudovirus	No data	1.2	No data		86.09	84.18
G339D, S371L, S373P, S375F, K417N, N440K, G446S, S477N, T478K, E484A, Q493R, G496S, Q498R, N501Y and Y505H	BA.1	Pseudovirus	1,013	< 5	183	No data	51.52	48.13
		Virus isolate	No data	< 5	30	No data		
G339D, S371L, S373P, S375F, K417N, N440K, G446S, S477N, T478K, E484A, Q493R, G496S, Q498R, N501Y and Y505H	BA.2	Pseudovirus	No data	16	3.2	No data	0.07	0.01
		Virus isolate	No data	15.7	5.4	No data		
G339D, S371L, S373P, S375F, K417N, N440K, G446S, S477N, T478K, E484A, Q493R, G496S, Q498R, N501Y and Y505H	BA.4	Pseudovirus	No data	21.3	65	No data	0.00	0.00
		Virus isolate	No data	48.4	No data		0.00	0.00
G339D, S371L, S373P, S375F, K417N, N440K, G446S, S477N, T478K, E484A, Q493R, G496S, Q498R, N501Y and Y505H	BA.5	Pseudovirus	No data	22.6	65	No data	0.00	0.00
		Virus isolate	No data	21.6	16	No data	0.00	0.00
G339D, S371L, S373P, S375F, K417N, N440K, G446S, S477N, T478K, E484A, Q493R, G496S, Q498R, N501Y and Y505H	BA.2.75	Pseudovirus	No data	8.3	15	No data	0.00	0.00
G339D, S371F, S373P, S375F, T376A, D405N, R408S, K417N, N440K, L452R, S477N, T478K, E484A, F486V, Q498R, N501Y, Y505H, K444T, N460K	BQ.1	Pseudovirus	No data	28.5	> 2,000	No data	77.55	75.6
G339D, S371F, S373P, S375F, T376A, D405N, R408S, K417N, N440K, L452R, S477N, T478K, E484A, F486V, Q498R, N501Y, Y505H, R346T, K444T and N460K	BQ.1.1	Pseudovirus	No data	94	> 2,000	No data	76.94	75.77
G339H, R346T, L368I, S371F, S373P, S375F, T376A, D405N, R408S, K417N, N440K, V445P, G446S, N460K, S477N, T478K, E484A, F486P, F490S, Q498R, N501Y and Y505H	XBB	Pseudovirus	No data	6.5	1,400	No data	21.76	22.06
G339H, R346T, L368I, S371F, S373P, S375F, T376A, D405N, R408S, K417N, N440K, V445P, G446S, N460K, S477N, T478K, E484A, F486P, F490S, Q498R, N501Y and Y505H	XBB.1.5	Pseudovirus	No data	11.3	> 5,000	No data	74.15	72.26
T19I, R21T, L24, P25, P26, A27S, S50L, H69, V70, V127F, G142D, Y144, F157S, R158G, N211, L212I, V213G, L216F, H245N, A264D, I332V, G339H, K356T, S371F, S373P, S375F, T376A, R403K, D405N, R408S, K417N, N440K, V445H, G446S, N450D, L452W, N460K, S477N, T478K, N481K, V483, E484K, F486P, Q498R, N501Y, Y505H, E554K, A570V, D614G, P621S, H655Y, I670V, N679K, P681R, N764K, D796Y, S939F, Q954H, N969K and P1143L	BA.2.86	Pseudovirus	No data		> 5,000	No data	0.01	0.00
T19I, R21T, L24, P25, P26, A27S, S50L, H69, V70, V127F, G142D, Y144, F157S, R158G, N211, L212I, V213G, L216F, H245N, A264D, I332V, G339H, K356T, S371F, S373P, S375F, T376A, R403K, D405N, R408S, K417N, N440K, V445H, G446S, N450D, L452W, L455S, N460K, S477N, T478K, N481K, V483, E484K, F486P, Q498R, N501Y, Y505H, E554K, A570V, D614G, P621S, H655Y, I670V, N679K, P681R, N764K, D796Y, S939F, Q954H, N969K and P1143L	JN.1	Pseudovirus	No data	No data	> 5,000	No data	0.01	0.00

EU/EEA: European Union or European Economic Area; GISAID: Global Initiative on Sharing All Influenza Data; SARS-CoV-2: severe acute respiratory syndrome coronavirus 2.

^a^ Monoclonal antibodies approved by the European Medicines Agency.

^b^ Global and EU/EEA frequency in percentage among sequenced isolates deployed to GISAID. Please refer to methods for the analysed time period and calculation of frequencies.

No data means no published data were available in the period of compiling and analysing the data.

The combination of mutations in BQ.1 (G339D, S371F, S373P, S375F, T376A, D405N, R408S, K417N, N440K, L452R, S477N, T478K, E484A, F486V, Q498R, N501Y, Y505H, K444T and N460K) and the mutations in the BQ.1.1 sub-lineage (G339D, S371F, S373P, S375F, T376A, D405N, R408S, K417N, N440K, L452R, S477N, T478K, E484A, F486V, Q498R, N501Y, Y505H, R346T, K444T and N460K) resulted in > 2,000-fold decreases in susceptibility to Evusheld [11]. This set of mutations within the respective sub-lineages was present in > 75% of the isolates (Table 2).

The set of mutations in the XBB and XBB.1.5 sub-lineages (G339H, R346T, L368I, S371F, S373P, S375F, T376A, D405N, R408S, K417N, N440K, V445P, G446S, N460K, S477N, T478K, E484A, F486P, F490S, Q498R, N501Y and Y505H) conferred 1,400 and > 5,000-fold reduction in susceptibility to Evusheld, respectively [24]. Further on, the unique sets of mutations in BA.2.86 and JN.1 shown in Table 2 correlated with high fold reductions (> 5,000) in neutralisation efficacy for Evusheld, however, their proportion was < 0.01%.

The mutations identified in ORF1ab, specifically nsp5:H172Y, nsp5:H172Y and Q189E, nsp5:L50F and E166V and nsp5:L50F, E166A and L167V, led to a decrease in susceptibility to Paxlovid (nirmatrelvir and ritonavir), ranging from moderate (25-99) to high (> 100) fold reductions [26,29-31]. Additionally, the mutation nsp12:S861G resulted in a moderate fold reduction in susceptibility to Veklury (remdesivir) [32] (Table 3).

**Table 3:** Mutations in the ORF1ab protein of SARS-CoV-2 and reductions in susceptibility to antiviral drugs in a review on the susceptibility of monoclonal antibodies and antiviral drugs targeted against SARS-CoV-2, April 2022–November 2024 (n = 30)^a^

Mutation (ORF1ab protein)	Reduction in susceptibility		Proportion	
	Veklury (remdesivir)	Paxlovid (nirmatrelvir and ritonavir)	Global^b^	EU/EEA
nsp5:E166A	No data	21.2	0.00	0.00
nsp5:F140L	No data	4.1	0.00	0.00
nsp5:H172Q	No data	42	0.00	0.00
nsp5:H172Y	No data	114	0.00	0.00
nsp5:L50F	No data	1.5	0.00	0.00
nsp5:Q189K	No data	6.3	0.00	0.00
nsp5:S144A	No data	20.5	0.00	0.00
nsp5:S144G	No data	15	0.00	0.00
nsp5:S144Y	No data	18	0.00	0.00
nsp5:Y54A	No data	23.6	0.00	0.00
nsp5:H172Y and Q189E	No data	281	0.00	0.00
nsp5:L50F and E166A	No data	27	0.00	0.00
nsp5:L50F and E166V	No data	300	0.00	0.00
nsp5:L50F, E166A and L167V	No data	29	0.00	0.00
nsp12:C799F	11.5	No data	0.00	0.00
nsp12:C799R	2.7	No data	0.00	0.00
nsp12:D484Y	3.1	No data	0.00	0.00
nsp12:E796G	2.6	No data	0.00	0.00
nsp12:E802A	3.9	No data	0.00	0.00
nsp12:E802D	7.3	No data	0.00	0.00
nsp12:F480L	3.8	No data	0.00	0.00
nsp12:N198S	10.4	No data	0.01	0.01
nsp12:S861G	29.7	No data	0.00	0.00
nsp12:S861A	2.8	No data	0.00	0.00
nsp12:V166A	10.4	No data	0.00	0.00
nsp12:V557L	5.7	No data	0.00	0.00
nsp12:V792I	8	No data	0.00	0.00
nsp12:F480L and V557L	3.8	No data	0.00	0.00
nsp12:V166A and L167V	10	No data	0.00	0.00
nsp12:L50F, V166A and L167V	1.4	No data	0.00	0.00

EU/EEA: European Union or European Economic Area; ORF: open reading frame; SARS-CoV-2: severe acute respiratory syndrome coronavirus 2.

^a^ Antiviral drugs approved by the European Medicines Agency.

^b^ Global and EU/EEA frequency in percentage among sequenced isolates deployed to GISAID. Please refer to methods for the calculation of frequencies.

No data means no data were available when the data were compiled and analysed.

The data used for calculating the median fold reduction values for the neutralisation susceptibility of the four mAbs are shown in Supplementary Table 2. The data extracted revealed highly decreased neutralisation capabilities of Ronapreve (casirivimab and imdevimab) across all Omicron sub-lineages, particularly evident in BA.1, BA.2.75 and XBB.1.5 (> 1,000, 911 and 615-fold reductions, respectively) (Table 4). Similar reduced susceptibility patterns were observed for Regkirona (regdanvimab), displaying substantial reduction (> 1,000-fold) in sub-lineages BA.1, BA.2 and BA.5. The findings indicated that Xevudy (sotrovimab) exhibited high neutralisation efficacy against most SARS-CoV-2 sub-lineages, however, a decrease in neutralisation activity was observed for BA.2, BA.4, BQ.1.1 and BA.2.86. Highest decrease in neutralisation susceptibility for sotrovimab was observed for BA.2. 86 (66-fold reduction). Evusheld (tixagevimab and cilgavimab) displayed highly reduced neutralisation activity against the BQ.1, BQ.1.1, XBB, XBB.1.5 and BA.2.86 sub-lineages (476, 476, 299, 863, 593-fold-reduction, respectively).

**Table 4:** Median fold reduction values for neutralisation susceptibility of monoclonal antibodies to 14 variants and sub-lineages of SARS-CoV-2, review on the susceptibility of monoclonal antibodies and antiviral drugs targeted against SARS-CoV-2, April 2022–November 2024 (n = 14)^a^

Variant or sub-lineage	Ronapreve (casirivimab and imdevimab)	Xevudy (sotrovimab)	Evusheld (tixagevimab and cilgavimab)	Regkirona (regdanvimab)
Alpha	0.9	1.8	0.8	1.2
Beta	1.6	1	1.7	39
Delta	2.2	1.5	1.1	8.6
Gamma	0.9	1.4	0.8	99.5
BA.1	> 1,000	4.8	81	> 1,000
BA.2	164	29	7.8	> 1,000
BA.2.75	911.5	16	19.5	25
BA.2.86	No data	66	593	No data
BA.4	232.5	27	8	No data
BA.5	432	19	20	> 1,000
BQ.1	100.5	14.5	476	No data
BQ.1.1	200	45	476	No data
XBB	100.5	8.1	299	No data
XBB.1.5	615	6.3	863	No data

SARS-CoV-2: severe acute respiratory syndrome coronavirus 2.

^a^ Monoclonal antibodies approved by the European Medicines Agency.

No data means no published data were available when the data were compiled and analysed.

## Discussion

The continuous emergence of new variants of SARS-CoV-2, some of which possess altered antigenic characteristics, may lead to resistance against current antiviral drugs and/or mAbs and evade immunity induced naturally or by vaccination. Such developments could have considerable implications for treatment and public health.

Expediting the assessment of resistance patterns in new virus variants is critical for assessment of fitness and clinical and public health importance of the viruses. In this evaluation, we used established methods and techniques to ascertain the impact and importance of emerging variants in terms of their resistance to treatments. The mutations nsp5:H172Y, nsp5:H172Y and Q189E, nsp5:L50F and E166V and nsp5:L50F, E166A and L167V in the Mpro protein are concerning due to their role in causing either partial or complete resistance to Paxlovid (nirmatrelvir and ritonavir) [26,29-31]. This review demonstrated that certain mutations, such as E337R/L and E340K/V/A in the SARS-CoV-2 Delta variant [28] and the combination of K417N, E484K and N501Y in the Beta variant, resulted in highly reduced neutralisation by Regkirona [23]. The combination of K417N, E484K and N501Y mutations in the Beta variant was common (80.4% globally and 83.4% in the EU/EEA countries), suggesting a more important role in reducing the efficacy of Regkirona during the period of Beta circulation. Similarly, the mutation sets found in the BQ.1 and BQ.1.1 sub-lineages, which caused > 2,000-fold reductions in susceptibility to Evusheld [11], were present in > 75% of the isolates. This highlights the importance of monitoring mutation frequencies in real time, as highly impactful mutations, even if they confer considerable resistance to mAbs, will have varying clinical relevance depending on how widely those mutations are distributed within the population. Additionally, sub-lineages BA.4 and BA.5 harbouring the same set of mutations showed moderately reduced neutralisation efficacy for Evusheld, emphasising that the context of specific variants and their mutation frequencies can lead to different levels of therapeutic efficacy [11]. This underscores the necessity for ongoing surveillance to track the prevalence of these critical mutations, allowing for timely adjustments to treatment strategies as viral variants evolve.

In vitro studies indicated that Ronapreve (casirivimab and imdevimab) had lost its efficacy against all SARS-CoV-2 Omicron sub-lineages [33-35], although it effectively neutralised the Alpha, Beta and Delta variants. A randomised double-blind placebo trial revealed that a single dose of casirivimab and imdevimab prevented symptomatic SARS-CoV-2 infections by 81% in generally healthy household contacts of SARS-CoV2-infected individuals, in pre-Omicron lineage variants [36]. Regkirona (regdanvimab) displayed highly reduced neutralising activity against Omicron sub-lineages BA.1, BA.2 and BA.5, resulting in complete resistance in vitro [37]. Prior to the emergence of the Omicron variant, clinical trials demonstrated that Regkirona (regdanvimab) notably decreased the number of patients progressing to severe or critical COVID-19 conditions and shortened recovery [23,38]. Xevudy (sotrovimab) retained full neutralising effect against most SARS-CoV-2 variants. Nonetheless, it exhibited reduced activity against specific Omicron sub-lineages, such as BA.2, BA.4 and BQ.1.1, in neutralisation tests in cell cultures, but not in studies in hamsters [39,40]. Further on, Xevudy (sotrovimab) maintained its in vitro effector functions against BA.2 and provided a Fc-dependant protection in the lungs of mice infected with BA.2 [41], aligning with the observed low hospitalisation and mortality rates among patients treated with Xevudy (sotrovimab) during the BA.2 and BA.5 waves [42,43]. This discrepancy warrants further investigation and may require additional tests to clarify the observed differences. A lineage different from XBB, designated as BA.2.86, emerged in August 2023, and during the summer of 2024 its median proportion ranged between 95-100% in the EU/EEA countries. This variant exhibited > 30 mutations in the S protein, i.e. more than both XBB and the parental BA.2. Many of these mutations were assumed to be linked to the evasion of the immune response and possible reductions in susceptibility to different mAbs [44]. Indeed, our analysis confirmed the assumption and showed a decreased neutralising efficacy of Xevudy (sotrovimab) against the BA.2.86 sub-lineage. Furthermore, of all the published literature so far, a unanimous conclusion can be drawn that Xevudy (sotrovimab) did not exhibit antiviral effects against BA.2.86 [44-46].

Evusheld (tixagevimab and cilgavimab) showed highly reduced neutralising activity against the BQ.1, BQ.1.1 and BA.2.86 sub-lineages, however, data for BA.2.86 were limited to only one study included in the analysis [11,44]. Concerns regarding treatment failure due to the emergence of Evusheld-resistant viral variants prompted a statement from the FDA [11]. Virus-like particles (VLPs) pseudotyped with the S protein from Omicron sub-lineages BQ.1 or BQ.1.1 demonstrated > 2,000-fold reductions in neutralising activity [11].

However, the study is limited by the uncertainty regarding how the neutralisation data from pseudotyped VLPs or virus isolates correlate with clinical outcomes in patients. Monitoring the resistance of emerging variants to mAb-based antiviral treatments is crucial in determining whether certain mAbs should be phased out or if different combinations of mAbs need to be considered. The emergence of drug-resistant variants can compromise the effectiveness of antiviral drugs, thus information gathered from antiviral drug resistance monitoring can help in updating treatment guidelines, modifying drug combinations or developing new antiviral drugs to stay ahead of emerging resistant variants.

A considerable variability in neutralisation susceptibility data exists, resulting in discordant findings across different investigations [47,48]. This variability arises due to the utilisation of diverse neutralising assays, such as those conducted in cell culture using clinical isolates or chimeric, recombinant and pseudotyped viruses. The results obtained for a specific specimen against a particular virus variant could differ between laboratories using the same type of assay owing to variations in the quantity of the virus inoculum and the cells employed for culture [47,48]. The variability in neutralisation susceptibility data can introduce uncertainty and challenges the interpretation of the results, which may have ultimately influenced the outcomes of our study. Expectations point towards enhanced reproducibility across studies as neutralising assays become more standardised, especially with the increased utilisation of external controls provided by the WHO [49].

## Conclusion

Data on the efficacy of mAbs and antivirals against SARS-CoV-2 are continuously increasing. By integrating antiviral resistance data with ongoing surveillance of SARS-CoV-2 variants, we can track the emergence and spread of drug-resistant strains and guide treatment strategies, such as phasing out ineffective mAbs, optimising mAb combinations, and updating guidelines, thus mitigating the public health risks posed by these variants.

Together, ECDC and WHO/Europe constantly monitor the circulation of SARS-CoV-2 variants through data reported to them from primary and secondary care sentinel and non-sentinel sources. Leveraging on the existing system for influenza antiviral resistance monitoring, it might be beneficial to establish a similar global system for monitoring antiviral resistance of SARS-CoV-2. This would involve defining criteria for assessing antiviral susceptibility and gathering clinical information to correlate with laboratory findings for COVID-19 therapeutics.

## Supplementary Data

558000 Supplementary Material
