# Supplementary material for: Impact of SARS-CoV-2 variant mutations on susceptibility to monoclonal antibodies and antiviral drugs: a non-systematic review, April 2022 to October 2024
Source: Euro Surveill. 2025 Mar 13;30(10):2400252. doi: 10.2807/1560-7917.ES.2025.30.10.2400252 (PMC11912142; doi:10.2807/1560-7917.ES.2025.30.10.2400252)
Supplement: Supplementary Material [file 24-00252_BROBERG_Supplement.pdf]

This supplementary material is hosted by Eurosurveillance as supporting information alongside the article "Impact of SARS-CoV-2 variant mutations on susceptibility to antiviral drugs and monoclonal antibodies: a non-systematic review, April 2022 to October 2024", on behalf of the authors, who remain responsible for the accuracy and appropriateness of the content. The same standards for ethics, copyright, attributions and permissions as for the article apply. Supplements are not edited by Eurosurveillance and the journal is not responsible for the maintenance of any links or email addresses provided therein.

Supplemental table 1.

Individual and combination of SARS-CoV-2 spike mutations for which data were available.

Antiviral drug/mAb & variant	Gene	AA replacement	Fold change	Reference
Veklury (remdesivir)	ORF1ab	E802D	2.5-7.3	[3]<br>[4]
	ORF1ab	F480L+V557L	3.8	[3]
	ORF1ab	E796G	2.6	[3]
	ORF1ab	C799F	1.9-11.5	[7]<br>[3]
	ORF1ab	D484Y	3.1	[3]
	ORF1ab	F480L	1.5-3.8	[10]<br>[3]
	ORF1ab	V557L	3.8-5.7	[10]<br>[3]
	ORF1ab	E802A	2.1-3.9	[3]<br>[4]
	ORF1ab	V792I	2.6	[7]
	ORF1ab	C799R	2.7	[7]
	ORF1ab	V166A	10.4	[7]
	ORF1ab	V166A+L167V	10	[16]
	ORF1ab	L50F+V166A+L167V	1.4	[16]
	ORF1ab	N198S	10.4	[7]
	ORF1ab	S861G	29.7	[7]
	ORF1ab	S861A	2.8	[7]

ORF1ab	L50F	1.5	[16]
ORF1ab	Y54A	23.6	[21]
ORF1ab	S144A	8	[23]
ORF1ab	S144A	20.5	[24]
ORF1ab	S144G	15	[24]
ORF1ab	S144Y	18.1	[24]
ORF1ab	S144F	25.1	[24]
ORF1ab	F140L	4.1	[21]
ORF1ab	E166A	16.4	[24]
ORF1ab	E166A	21.2	[23]
ORF1ab	H172Y	24.5	[25]
ORF1ab	H172Y	114	[24]
ORF1ab	H172Q	42	[24]
ORF1ab	Q189K	6.3	[24]
ORF1ab	L50F+E166A+L167V	29	[16]
ORF1ab	L50F+E166A	27	[23]
ORF1ab	L50F+E166V	300	[23]

	ORF1ab	H172Y+Q189E	281.1	[24]
Ronapreve (casirivimab / imdevimab) Alpha (B.1.1.7)	S	N501Y	<2	[5]
Evusheld (tixagevimab / cilgavimab) Alpha (B.1.1.7)	S	N501Y	1.0 - 5.2	[8]
	S	N501Y	0.5 - 1.4	[8]
	S	N501Y	0.5-5.2	[8]
Regkirona (regdanvimab) Alpha (B.1.1.7)	S	N501Y+P681H	<5	[11]
	S	N501Y+P681H	<5	[11]
Ronapreve (casirivimab / imdevimab) Beta (B.1.351)	S	K417N+E484K+N501Y	<2	[5]
Evusheld (tixagevimab / cilgavimab) Beta (B.1.351)	S	K417N+E484K+N501Y	2.5 - 5.5	[8]
	S	K417N+E484K+N501Y	0.9 - 3.8	[8]
	S	K417N+E484K+N501Y	<5	[9]
	S	K417N+E484K+N501Y	<5	[9]
Regkirona (regdanvimab) Beta (B.1.351)	S	K417N+E484K+N501Y	184.3	[11]
	S	K417N+E484K+N501Y	19.7	[11]
Xevudy (sotrovimab) Gamma (P.1)	S	K417T+E484K+N501Y	<5	[1]
Evusheld (tixagevimab / cilgavimab) Gamma (P.1)	S	K417T+E484K+N501Y	0.8 - 1.7	[8]
	S	K417T+E484K+N501Y	0.4 - 2.0	[8]
	S	K417T+E484K+N501Y	<5	[9]
	S	K417T+E484K+N501Y	<5	[9]
Regkirona (regdanvimab) Gamma (P.1)	S	K417T + E484K + N501Y	61.4	[11]
	S	K417T + E484K + N501Y	137.9	[11]
Xevudy (Sotrovimab) Delta (B.1.617.2)	S	P337L	192	[2]
Xevudy (Sotrovimab) Delta (B.1.617.2)	S	P337R	192	[2]
Xevudy (Sotrovimab) Delta (B.1.617.2)	S	P337T	11	[2]
Xevudy (Sotrovimab) Delta (B.1.617.2)	S	P337H	5	[2]
Xevudy (Sotrovimab) Delta (B.1.617.2)	S	E340K	297	[2]

Xevudy (Sotrovimab) Delta (B.1.617.2)	S	E340K	297	[1]
Xevudy (Sotrovimab) Delta (B.1.617.2)	S	E340V	200	[1] [2]
Xevudy (Sotrovimab) Delta (B.1.617.2)	S	E340Q	50	[2]
Xevudy (Sotrovimab) Delta (B.1.617.2)	S	E340G	18	[2]
Ronapreve (casirivimab / imdevimab) Delta (B.1.617.2)	S	L452R+T478K	<2	[5]
	S	L452R+T478K	<2	[6]
Evusheld (tixagevimab / cilgavimab) Delta (B.1.617.2)	S	L452R+T478K	1 - 1.2	[8]
	S	L452R+T478K	0.6 - 1.0	[8]
	S	L452R+T478K	<5	[9]
	S	L452R+T478K	<5	[9]
Regkirona (regdanvimab) Delta (B.1.617.2)	S	K417N+L452R+T478K	27.7	[11]
	S	K417N+L452R+T478K	183	[11]
Ronapreve (casirivimab / imdevimab) Omicron BA.1	S	G339D+S371L+S373P+S375F+ K417N+N440K+G446S+S477N+ T478K+ E484A+Q493R+G496S+ Q489R+N501Y+Y505H	1013	[5]
	S	G339D	1.8	[12]
	S	S373P	3	[13]
	S	S375F	0.8	[14]
	S	K417N	2.5	[15]
	S	N440K	2.25	[17]
	S	G446S	3.3	[18]
	S	E484A	4.3	[19]
Xevudy (sotrovimab) Omicron BA.1	S	G339D+S371L+S373P+S375F+ K417N+N440K+G446S+S477N+ T478K+ E484A+Q493R+G496S+ Q489R+N501Y+Y505H	<5	[1]
	S	G339D+S371L+S373P+S375F+ K417N+N440K+G446S+S477N+ T478K+ E484A+Q493R+G496S+ Q489R+N501Y+Y505H	<5	[1]
	S	G339D	2	[22]
Evusheld (tixagevimab / cilgavimab) Omicron BA.1	S	G339D+S371L+S373P+ S375F+K417N+N440K+ G446S+S477N+T478K+ E484A+Q493R+G496S+ Q489R+N501Y+Y505H	132- 183	[9]

	S	G339D+S371L+S373P+ S375F+K417N+N440K+ G446S+S477N+T478K+ E484A+Q493R+G496S+ Q489R+N501Y+Y505H	12-30	[8]
Evusheld (tixagevimab / cilgavimab) Omicron BA.2	S	G339D+ S371F+ S373P+ S375F+ T376A+ D405N+ R408S+ K417N+ N440K+ S477N+ T478K+E484A+ Q493R+ Q498R+ N501Y+ Y505H+ H655Y+ N679K+ P681H+ N764K	3.2	[8]
	S	G339D+ S371F+ S373P+ S375F+ T376A+ D405N+ R408S+ K417N+ N440K+ S477N+ T478K+E484A+ Q493R+ Q498R+ N501Y+ Y505H+ H655Y+ N679K+ P681H+ N764K	5.4	[8]
	S	G339D+S371F+S373P+ S375F+T376A+D405N+ R408S+K417N+N440K+ S477N+T478K+E484A+ Q493R+Q498R+N501Y+ Y505H	5.4	[9]
Xevudy (sotrovimab) Omicron BA.2	S	G339D+S371L+S373P+S375F+K417N+N440K+G446S+S477N+T478K+E484A+Q493R+G496S+ Q489R+N501Y+ Y505H	16	[1]
Xevudy (sotrovimab) Omicron BA.2	S	G339D+S371L+S373P+S375F+K417N+N440K+G446S+S477N+T478K+E484A+Q493R+G496S+ Q489R+N501Y+ Y505H	15.7	[1]
Evusheld (tixagevimab / cilgavimab) Omicron BA.4	S	G339D+S371F+S373P+ S375F+T376A+D405N+ R408S+K417N+N440K+ S477N+T478K+E484A+ Q493R+Q498R+N501Y+ Y505H	33 - 65	[9]
Xevudy (sotrovimab) Omicron BA.4	S	G339D+S371L+S373P+S375F+K417N+N440K+G446S+S477N+T478K+E484A+Q493R+G496S+ Q489R+ N501Y+ Y505H	21.3	[1]
Xevudy (sotrovimab) Omicron BA.4	S	G339D+S371L+S373P+S375F+K417N+N440K+G446S+S477N+T478K+E484A+Q493R+G496S+ Q489R+ N501Y+ Y505H	48.4	[1]
Evusheld (tixagevimab / cilgavimab) Omicron BA.5	S	G339D+S371F+S373P+ S375F+T376A+D405N+ R408S+K417N+N440K+ S477N+T478K+E484A+ Q493R+Q498R+N501Y+ Y505H	33 – 65	[9]
	S	G339D+S371F+S373P+ S375F+T376A+D405N+ R408S+K417N+N440K+ S477N+T478K+E484A+ Q493R+Q498R+N501Y+ Y505H	4.2 – 16	[8]
	S	G339D+S371F+S373P+ S375F+T376A+D405N+ R408S+K417N+N440K+ S477N+T478K+E484A+ Q493R+Q498R+N501Y+ Y505H	33-65	[8]
Xevudy (sotrovimab) Omicron BA.5	S	G339D+S371L+S373P+S375F+K417N+N440K+G446S+S477N+T478K+E484A+Q493R+G496S+ Q489R+ N501Y+ Y505H	22.6	[1]
Xevudy (sotrovimab) Omicron BA.5	S	G339D+S371L+S373P+S375F+K417N+N440K+G446S+S477N+T478K+E484A+Q493R+G496S+ Q489R+ N501Y+ Y505H	21.6	[1]
Evusheld (tixagevimab / cilgavimab) Omicron BA.2.75	S	G339H+S371F+S373P+ S375F+T376A+D405N+ R408S+K417N+N440K+ G446S+N460K+S477N+ T478K+E484A+Q498R+ N501Y+ Y505H	2.4-15	[9]
Xevudy (sotrovimab) Omicron BA.2.75	S	G339D+S371L+S373P+S375F+K417N+N440K+G446S+S477N+T478K+E484A+Q493R+G496S+ Q489R+ N501Y+ Y505H	8.3	[1]
Evusheld		G339D+S371F+S373P+		

(tixagevimab / cilgavimab) Omicron BQ.1	S	S375F+T376A+D405N+ R408S+K417N+N440K+ L452R+S477N+T478K+ E484A+F486V+Q498R+ N501Y+Y505H+K444T+N460K	>2000	[9]
Xevudy (sotrovimab) Omicron BQ.1	S	G339D+S371F+S373P+S375F+T376A+D405N+R408S+K417N+N440K+L452R+S477N+T478K+E484A+F486V+Q498R+N501Y+Y505H+K444T+N460K	28.5	[1]
Evusheld (tixagevimab / cilgavimab) Omicron BQ.1.1	S	G339D+S371F+S373P+ S375F+T376A+D405N+ R408S+K417N+N440K+ L452R+S477N+T478K+ E484A+F486V+Q498R+ N501Y+Y505H+R346T+K444T+ N460K	>2000	[9]
Xevudy (sotrovimab) Omicron BQ.1.1	S	G339D+S371F+S373P+S375F+T376A+D405N+R408S+K417N+N440K+L452R+S477N+T478K+E484A+F486V+Q498R+N501Y+Y505H+K444T+N460K	94	[1]
Evusheld (tixagevimab/ cilgavimab) Omicron XBB	S	G339H+R346T+L368I+S371F+S373P+S375F+T376A+D405N+R408S+ K417N+N440K+V445P+G446S+N460K+S477N+T478K+E484A+F486P+ F490S+Q498R+N501Y +Y505H	1400	[8]
Xevudy (sotrovimab) Omicron XBB	S	G339H+R346T+L368I+S371F+S373P+S375F+T376A+D405N+R408S+ K417N+N440K+V445P+G446S+N460K+S477N+T478K+E484A+F486P+ F490S+Q498R+N501Y +Y505H	6.5	[1]
Xevudy (sotrovimab) Omicron XBB 1.5	S	G339H+R346T+L368I+S371F+S373P+S375F+T376A+D405N+R408S+K417N+N440K+V445P+G446S+N460K+S477N+T478K+E484A+F486P+F490S+Q498R+N501Y +Y505H	11.3	[1]
Evusheld (tixagevimab/ cilgavimab) Omicron XBB 1.5	S	G339H+R346T+L368I+S371F+S373P+S375F+T376A+D405N+R408S+K417N+N440K+V445P+G446S+N460K+S477N+T478K+E484A+F486P+F490S+Q498R+N501Y +Y505H	>5000	[8]
Evusheld (tixagevimab/ cilgavimab) Omicron BA.2.86	s	T19I+R21T+L24-+P25- +P26- +A27S +S50L+ H69- +V70-+V127F+ G142D+ Y144-+F157S+ R158G+ N211- +L212I+ V213G+L216F+H245N +A264D+I332V+G339H+ K356T+S371F+S373P+ S375F+T376A+R403K+ D405N+R408S+K417N+ N440K+V445H+G446S+ N450D+L452W+N460K+ S477N+T478K+N481K+ V483-+E484K+F486P+ Q498R+N501Y+Y505H+ E554K+A570V+D614G+ P621S+H655Y+I670V+ N679K+P681R+N764K+ D796Y+S939F+Q954H+ N969K+P1143L	>5000	[8]
Evusheld (tixagevimab/ cilgavimab) Omicron JN.1	s	T19I+R21T+L24-+P25-+P26- +A27S+S50L+ H69-+V70-+V127F+G142D +Y144-+F157S+R158G+ N211-+L212I+V213G+ L216F+H245N+A264D+ I332V+G339H+K356T+ S371F+S373P+S375F+ T376A+R403K+D405N+ R408S+K417N+N440K+ V445H+G446S+N450D+ L452W+L455S+N460K+ S477N+T478K+N481K+ V483-+E484K+F486P+	>5000	[8]

		Q498R+N501Y+Y505H+ E554K+A570V+D614G+ P621S+H655Y+I670V+ N679K+P681R+N764K+ D796Y+S939F+Q954H+ N969K+P1143L		

References

1. Food and Drug Administration (FDA). FACT SHEET FOR HEALTHCARE PROVIDERS: EMERGENCY USE AUTHORIZATION FOR SOTROVIMAB Silver Springs: FDA; 2023. Available at: https://www.fda.gov/media/149534/download [Access date: 4 Nov 2024]

2. Cathcart AL, Havenar-Daughton C, Lempp FA, Ma D, Schmid MA, Agostini ML, et al. The dual function monoclonal antibodies VIR-7831 and VIR-7832 demonstrate potent in vitro and in vivo activity against SARS-CoV-2. bioRxiv. 2022:2021.03.09.434607. Available at: https://www.biorxiv.org/content/biorxiv/early/2022/04/01/2021.03.09.434607.full.pdf

3. Torii S, Kim KS, Koseki J, Suzuki R, Iwanami S, Fujita Y, et al. Characterization of various remdesivir-resistant mutations of SARS-CoV-2 by mathematical modeling and molecular dynamics simulation. bioRxiv. 2022:2022.02.22.481436. Available at: https://www.biorxiv.org/content/biorxiv/early/2022/02/24/2022.02.22.481436.full.pdf

4. Szemiel AM, Merits A, Orton RJ, MacLean OA, Pinto RM, Wickenhagen A, et al. In vitro selection of Remdesivir resistance suggests evolutionary predictability of SARS-CoV-2. PLoS Pathog. 2021 Sep;17(9):e1009929. Available at: https://www.ncbi.nlm.nih.gov/pubmed/34534263

5. Food and Drug Administration (FDA). FACT SHEET FOR HEALTH CARE PROVIDERS EMERGENCY USE AUTHORIZATION (EUA) OF REGEN-COV® (casirivimab and imdevimab). 2021 Available at: https://www.fda.gov/media/145611/download

6. European Medicines Agency (EMA). Ronapreve SUMMARY OF PRODUCT CHARACTERISTICS. 2022 Available at: https://www.ema.europa.eu/en/documents/product-information/ronapreve-epar-product-information_en.pdf

7. Stevens LJ, Pruijssers AJ, Lee HW, Gordon CJ, Tchesnokov EP, Gribble J, et al. Mutations in the SARS-CoV-2 RNA-dependent RNA polymerase confer resistance to remdesivir by distinct mechanisms. Sci Transl Med. 2022 Aug 3;14(656):eabo0718.

8. European Medicines Agency (EMA). Evusheld : EPAR - Product information. Amsterdam: EMA; 2024. Available at: https://www.ema.europa.eu/en/documents/product-information/evusheld-epar-product-information_en.pdf [Access date: 4 Nov 2024]

9. Food and Drug Administration (FDA). FACT SHEET FOR HEALTHCARE PROVIDERS: EMERGENCY USE AUTHORIZATION FOR EVUSHELD™ (tixagevimab co-packaged with cilgavimab) 2023 Available at: https://www.fda.gov/media/154701/download

10. Pruijssers AJ, George AS, Schäfer A, Leist SR, Gralinksi LE, Dinnon KH, 3rd, et al. Remdesivir Inhibits SARS-CoV-2 in Human Lung Cells and Chimeric SARS-CoV Expressing the SARS-CoV-2 RNA Polymerase in Mice. Cell Rep. 2020 Jul 21;32(3):107940.

11. European Medicines Agency (EMA). Regkirona SUMMARY OF PRODUCT CHARACTERISTICS. 2022 Available at: https://www.ema.europa.eu/en/documents/product-information/regkirona-epar-product-information_en.pdf

12. Shen X, Tang H, McDanal C, Wagh K, Fischer W, Theiler J, et al. SARS-CoV-2 variant B.1.1.7 is susceptible to neutralizing antibodies elicited by ancestral spike vaccines. Cell Host Microbe. 2021 Apr 14;29(4):529-39.e3.

13. Planas D, Veyer D, Baidaliuk A, Staropoli I, Guivel-Benhassine F, Rajah MM, et al. Reduced sensitivity of SARS-CoV-2 variant Delta to antibody neutralization. Nature. 2021 Aug;596(7871):276-80.

14. Yao W, Ma D, Wang H, Tang X, Du C, Pan H, et al. Effect of SARS-CoV-2 spike mutations on animal ACE2 usage and in vitro neutralization sensitivity. bioRxiv. 2021:2021.01.27.428353. Available at: https://www.biorxiv.org/content/biorxiv/early/2021/08/05/2021.01.27.428353.full.pdf

15. Liu C, Ginn HM, Dejnirattisai W, Supasa P, Wang B, Tuekprakhon A, et al. Reduced neutralization of SARS-CoV-2 B.1.617 by vaccine and convalescent serum. Cell. 2021 Aug 5;184(16):4220-36.e13.

16. Jochmans D, Liu C, Donckers K, Stoycheva A, Boland S, Stevens SK, et al. The Substitutions L50F, E166A, and L167F in SARS-CoV-2 3CLpro Are Selected by a Protease Inhibitor In Vitro and Confer Resistance To Nirmatrelvir. mBio. 2023 Feb 28;14(1):e0281522. Available at: https://www.ncbi.nlm.nih.gov/pubmed/36625640

17. Miyakawa K, Jeremiah SS, Kato H, Ryo A. Neutralizing efficacy of vaccines against the SARS-CoV-2 Mu variant. medRxiv. 2021:2021.09.23.21264014. Available at:

https://www.medrxiv.org/content/medrxiv/early/2021/09/26/2021.09.23.21264014.full.pdf
18. Wang R, Zhang Q, Ge J, Ren W, Zhang R, Lan J, et al. Analysis of SARS-CoV-2 variant mutations reveals neutralization escape mechanisms and the ability to use ACE2 receptors from additional species. Immunity. 2021 Jul 13;54(7):1611-21.e5.
19. Yuan M, Huang D, Lee CD, Wu NC, Jackson AM, Zhu X, et al. Structural and functional ramifications of antigenic drift in recent SARS-CoV-2 variants. bioRxiv. 2021 Feb 17 Available at: https://www.ncbi.nlm.nih.gov/pubmed/33619487
20. Rodriguez L, Lee H.W, Li J, Martin R, Han D, Xu S, et al. SARS-CoV-2 treatment: The impact of Remdesivir. Conference on Retroviruses and Opportunistic Infections (CROI): CROI Foundation/IAS-USA.; 2023. Available at: https://www.croiconference.org/abstract/remdesivir-resistance-analyses-from-the-pinetree-study-in-outpatients-with-covid-19/ [Access date: 11 Nov 2024]
21. Food and Drug Administration (FDA). FACT SHEET FOR HEALTHCARE PROVIDERS: EMERGENCY USE AUTHORIZATION FOR PAXLOVID2023. Available at: https://www.fda.gov/media/155050/download [Access date: 3 March 2025]
22. Food and Drug Administration. FDA authorizes revisions to fact sheets to address SARS-CoV-2 variants for monoclonal antibody products under emergency use authorization. Silver Springs: FDA; 2021. Available at: https://www.fda.gov/drugs/drug-safety-and-availability/fda-authorizes-revisions-fact-sheets-address-sars-cov-2-variants-monoclonal-antibody-products-under [Access date: 5 November 2024]
23. Moghadasi SA, Biswas RG, Harki DA, Harris RS. Rapid resistance profiling of SARS-CoV-2 protease inhibitors. npj Antimicrobials and Resistance. 2023 2023/08/20;1(1):9. Available at: https://doi.org/10.1038/s44259-023-00009-0
24. Hu Y, Lewandowski EM, Tan H, Zhang X, Morgan RT, Zhang X, et al. Naturally Occurring Mutations of SARS-CoV-2 Main Protease Confer Drug Resistance to Nirmatrelvir. ACS Central Science. 2023 2023/08/23;9(8):1658-69. Available at: https://doi.org/10.1021/acscentsci.3c00538
25. Clayton J, de Oliveira VM, Ibrahim MF, Sun X, Mahinthichaichan P, Shen M, et al. Integrative Approach to Dissect the Drug Resistance Mechanism of the H172Y Mutation of SARS-CoV-2 Main Protease. J Chem Inf Model. 2023 Jun 12;63(11):3521-33.

Supplemental table 2.

Variant	Gene	Protein	Antibody	Assay	Control (IC50 ng/ml)	IC50 (ng/ml)	Fold change	Reference
Alpha (B.1.1.7)	S	S	Ronapreve (casirivimab / imdevimab)	Virus isolate	Wild type	20	0.7	DOI: 10.1038/s41467-022-28766-y
	S	S	Ronapreve (casirivimab / imdevimab)	Virus isolate	Wild type	N/A	1	Regeneron EUA HCP Fact Sheet 01242022 (fda.gov)
	S	S	Ronapreve (casirivimab / imdevimab)	Pseudovirus (VSV)	Wild type (4.9)	21	4.3	https://www.ema.europa.eu/en/documents/referral/regn-cov2-antibody-combination-casirivimab/imdevimab-covid19-article-53-procedure-assessment-report_en.pdf
	S	S	Ronapreve (casirivimab / imdevimab)	Pseudovirus (VSV)	Wild type	1	1	DOI: 10.1016/j.cell.2021.03.036
	S	S	Ronapreve (casirivimab / imdevimab)	Pseudovirus (MLV)	Wild type	2	0.3	DOI: 10.1073/pnas.2205784119
	S	S	Ronapreve (casirivimab / imdevimab)	Pseudovirus (VSV)	Wild type	4.4	0.8	DOI: 10.1016/j.cell.2021.06.002
	S	S	Xevudy (sotrovimab)	Virus isolate	Wild type	78	2	DOI: 10.1016/j.cell.2021.12.046
	S	S	Xevudy (sotrovimab)	Virus isolate	Wild type	45	1.6	DOI: 10.1056/NEJMc2119407
	S	S	Xevudy (sotrovimab)	Pseudovirus (VSV)	Wild type	80	1.4	DOI: 10.1038/s41586-021-04385-3
	S	S	Xevudy (sotrovimab)	Pseudovirus (VSV)	Wild type	62	5.6	DOI: 10.1126/scitranslmed.abn6859
	S	S	Xevudy (sotrovimab)	Pseudovirus (lentivirus)	Wild type	9.2	192	DOI: 10.1016/j.chom.2021.03.002
	S	S	Xevudy (sotrovimab)	Pseudovirus (lentivirus)	Wild type	173	1.4	DOI: 10.1016/j.ebiom.2022.103944
	S	S	Xevudy (sotrovimab)	Pseudovirus (MLV)	Wild type	76	1.1	DOI: 10.1073/pnas.2205784119
	S	S	Xevudy (sotrovimab)	Pseudovirus	Wild type	N/A	1	Xevudy, INN-sotrovimab (europa.eu)
	S	S	Xevudy (sotrovimab)	Pseudovirus	Wild type	N/A	2.3	https://www.fda.gov/media/149534/download
	S	S	Xevudy (sotrovimab)	Pseudovirus (HIV)	Wild type	87	2.8	DOI: 10.1101/2022.01.30.478305
	S	S	Evusheld (tixagevimab / cilgavimab)	Virus isolate	Wild type	7	0.8	DOI: 10.1016/j.cell.2021.12.046
	S	S	Evusheld (tixagevimab / cilgavimab)	Virus isolate	Wild type	4	0.5	Evusheld, INN- tixagevimab, cilgavimab (europa.eu)

	S	S	Evusheld (tixagevimab / cilgavimab)	Pseudovirus (lentivirus)	Wild type	21	1.5	DOI: 10.1016/j.ebiom.2022.103944
	S	S	Evusheld (tixagevimab / cilgavimab)	Pseudovirus	Wild type	0.7	0.4	Evusheld, INN- tixagevimab, cilgavimab (europa.eu)
	S	S	Evusheld (tixagevimab / cilgavimab)	Pseudovirus	Wild type	N/A	2.9	https://www.fda.gov/media/154701/download
	S	S	Regkirona (regdanvimab)	Virus isolate	Wild type	2.8	1.4	DOI: 10.1016/j.bbrc.2021.06.016
	S	S	Regkirona (regdanvimab)	Virus isolate	Wild type	N/A	1	Regkirona, INN-regdanvimab (europa.eu)
	S	S	Regkirona (regdanvimab)	Pseudovirus (lentivirus)	Wild type	5.5	3.7	DOI: 10.1126/science.abn8897
	S	S	Regkirona (regdanvimab)	Pseudovirus	Wild type	N/A	1	Regkirona, INN-regdanvimab (europa.eu)
Beta (B.1.351)	S	S	Ronapreve (casirivimab / imdevimab)	Virus isolate	Wild type	0.8	5.1	DOI: 10.1093/cid/ciac143
	S	S	Ronapreve (casirivimab / imdevimab)	Virus isolate	Wild type	5.2	1.5	DOI: 10.1056/NEJMc2119407
	S	S	Ronapreve (casirivimab / imdevimab)	Virus isolate	Wild type	50	1.7	DOI: 10.1038/s41467-022-28766-y
	S	S	Ronapreve (casirivimab / imdevimab)	Virus isolate	Wild type	N/A	1	Regeneron EUA HCP Fact Sheet 01242022 (fda.gov)
	S	S	Ronapreve (casirivimab / imdevimab)	Virus isolate	Wild type	N/A	8.1	DOI: 10.1038/s41586-021-03398-2
	S	S	Ronapreve (casirivimab / imdevimab)	Pesudovirus (VSV)	Wild type	18	1.6	DOI: 10.1016/j.celrep.2022.110754
	S	S	Ronapreve (casirivimab / imdevimab)	Pesudovirus (VSV)	Wild type	1	1	DOI: 10.1016/j.cell.2021.03.036
	S	S	Ronapreve (casirivimab / imdevimab)	Pesudovirus (VSV)	Wild type	2.2	1	DOI: 10.1016/j.celrep.2021.109415
	S	S	Ronapreve (casirivimab / imdevimab)	Pesudovirus (HIV)	Wild type	15	9.1	DOI: 10.1128/mBio.00696-21
	S	S	Ronapreve (casirivimab / imdevimab)	Pseudovirus (MLV)	Wild type	20	3.3	DOI: 10.1073/pnas.2205784119
	S	S	Ronapreve (casirivimab / imdevimab)	Pseudovirus (VSV)	Wild type	N/A	1.3	DOI: 10.1038/s41586-021-03398-2

	S	S	Ronapreve (casirivimab / imdevimab)	Pseudovirus (lentivirus)	Wild type	129	5.7	DOI: 10.1101/2022.07.29.502029
	S	S	Ronapreve (casirivimab / imdevimab)	Pseudovirus (VSV)	Wild type	4.1	0.7	DOI: 10.1016/j.cell.2021.06.002
	S	S	Xevudy (sotrovimab)	Virus isolate	Wild type	101	3.7	DOI: 10.1056/NEJMc2119407
	S	S	Xevudy (sotrovimab)	Virus isolate	Wild type	82	2.1	DOI: 10.1016/j.cell.2021.12.046
	S	S	Xevudy (sotrovimab)	Virus isolate	Wild type	N/A	1	Xevudy, INN-sotrovimab (europa.eu)
	S	S	Xevudy (sotrovimab)	Virus isolate	Wild type	N/A	1.2	https://www.fda.gov/media/149534/download
	S	S	Xevudy (sotrovimab)	Pseudovirus (VSV)	Wild type	50	0.9	DOI: 10.1038/s41586-021-04385-3
	S	S	Xevudy (sotrovimab)	Pseudovirus (VSV)	Wild type	26	2.4	DOI: 10.1126/scitranslmed.abn6859
	S	S	Xevudy (sotrovimab)	Pseudovirus (VSV)	Wild type	606	4.9	DOI: 10.1016/j.ebiom.2022.103944
	S	S	Xevudy (sotrovimab)	Pseudovirus (lentivirus)	Wild type	28	0.8	DOI: 10.1126/science.abn8897
	S	S	Xevudy (sotrovimab)	Pseudovirus (lentivirus)	Wild type	192	0.1	DOI: 10.1101/2022.02.06.479332
	S	S	Xevudy (sotrovimab)	Pseudovirus (VSV)	Wild type	62	0.9	DOI: 10.1038/s41586-022-04466-x
	S	S	Xevudy (sotrovimab)	Pseudovirus (MLV)	Wild type	233	3.2	DOI: 10.1073/pnas.2205784119
	S	S	Xevudy (sotrovimab)	Pseudovirus (HIV)	Wild type	2	0.5	DOI: 10.1016/j.immuni.2021.06.003
	S	S	Xevudy (sotrovimab)	Pseudovirus (lentivirus)	Wild type	356	0.6	DOI: 10.1101/2022.07.29.502029
	S	S	Xevudy (sotrovimab)	Pseudovirus	Wild type	N/A	0.6	https://www.fda.gov/media/149534/download
	S	S	Xevudy (sotrovimab)	Pseudovirus (HIV)	Wild type	41	1.3	DOI: 10.1101/2022.01.30.478305
	S	S	Evusheld (tixagevimab / cilgavimab)	Virus isolate	Wild type	10	3	DOI: 10.1056/NEJMc2119407
	S	S	Evusheld (tixagevimab / cilgavimab)	Virus isolate	Wild type	12	1.3	DOI: 10.1016/j.cell.2021.12.046
	S	S	Evusheld (tixagevimab / cilgavimab)	Virus isolate	Wild type	6.5	0.8	Evusheld, INN- tixagevimab, cilgavimab (europa.eu)

	S	S	Evusheld (tixagevimab / cilgavimab)	Virus isolate	Wild type	N/A	1	https://www.fda.gov/media/154701/download
	S	S	Evusheld (tixagevimab / cilgavimab)	Pseudovirus (lentivirus)	Wild type	28	2	DOI: 10.1016/j.ebiom.2022.103944
	S	S	Evusheld (tixagevimab / cilgavimab)	Pseudovirus	Wild type	5.6	2.9	Evusheld, INN- tixagevimab, cilgavimab (europa.eu)
	S	S	Evusheld (tixagevimab / cilgavimab)	Pseudovirus (MLV)	Wild type	7	1.4	DOI: 10.1073/pnas.2205784119
	S	S	Evusheld (tixagevimab / cilgavimab)	Pseudovirus (lentivirus)	Wild type	29	2	DOI:10.1101/2022.07.29.502029
	S	S	Regkirona (regdanvimab)	Virus isolate	Wild type	40	20	DOI: 10.1016/j.bbrc.2021.06.016
	S	S	Regkirona (regdanvimab)	Virus isolate	Wild type	*N/A*	310	https://www.ema.europa.eu/en/documents/product-information/regkirona-epar-product-information_en.pdf
	S	S	Regkirona (regdanvimab)	Pseudovirus (HIV)	Wild type	330	33	DOI: 10.1016/j.bbrc.2021.06.016
	S	S	Regkirona (regdanvimab)	Pseudovirus (lentivirus)	Wild type	66	44	DOI: 10.1126/science.abn8897
	S	S	Regkirona (regdanvimab)	Pseudovirus	Wild type	*N/A*	184	Regkirona, INN-regdanvimab (europa.eu)
	S	S	Regkirona (regdanvimab)	Pseudovirus (lentivirus)	Wild type	647	34	DOI: 10.1101/2022.07.29.502029
Gamma (P.1)	S	S	Ronapreve (casirivimab / imdevimab)	Virus isolate	Wild type	2.1	0.6	DOI: 10.1056/NEJMc2119407
	S	S	Ronapreve (casirivimab / imdevimab)	Virus isolate	Wild type	20	0.7	DOI: 10.1038/s41467-022-28766-y
	S	S	Ronapreve (casirivimab / imdevimab)	Pseudovirus (HIV)	Wild type	27	16	DOI: 10.1128/mBio.00696-21
	S	S	Ronapreve (casirivimab / imdevimab)	Pseudovirus (MLV)	Wild type	17	2.8	DOI: 10.1073/pnas.2205784119
	S	S	Ronapreve (casirivimab / imdevimab)	Pseudovirus (VSV)	Wild type	4	0.7	DOI: 10.1016/j.cell.2021.06.002
	S	S	Ronapreve (casirivimab / imdevimab)	Pseudovirus (VSV)	Wild type	1	1	DOI: 10.1016/j.cell.2021.03.036
	S	S	Xevudy (sotrovimab)	Virus isolate	Wild type	21	0.5	DOI: 10.1126/scitranslmed.abn6859
	S	S	Xevudy (sotrovimab)	Pseudovirus (VSV)	Wild type	24	2.2	DOI: 10.1126/scitranslmed.abn6859

	S	S	Evusheld (tixagevimab / cilgavimab)	Virus isolate	Wild type (3.4)	1.8	0.5	DOI: 10.1056/NEJMc2119407
	S	S	Evusheld (tixagevimab / cilgavimab)	Virus isolate	Wild type (9)	6	0.7	DOI: 10.1016/j.cell.2021.12.046
	S	S	Evusheld (tixagevimab / cilgavimab)	Virus isolate	Wild type (8)	3.2	0.4	Evusheld, INN- tixagevimab, cilgavimab (europa.eu)
	S	S	Evusheld (tixagevimab / cilgavimab)	Pseudovirus	Wild type (1.9)	1.8	0.9	Evusheld, INN- tixagevimab, cilgavimab (europa.eu)
	S	S	Evusheld (tixagevimab / cilgavimab)	Pseudovirus (MLV)	Wild type (5)	4	0.8	DOI: 10.1073/pnas.2205784119
	S	S	Evusheld (tixagevimab / cilgavimab)	Pseudovius	Wild type	N/A	1.2	https://www.fda.gov/media/154701/download
	S	S	Regkirona (regdanvimab)	Virus isolate	Wild type (2)	276	138	DOI: 10.1016/j.bbrc.2021.09.023
	S	S	Regkirona (regdanvimab)	Virus isolate	Wild type	N.A.	168	Regkirona, INN-regdanvimab (europa.eu)
	S	S	Regkirona (regdanvimab)	Psedovirus (lentivirus)	Wild type (1.5)	40	26	DOI: 10.1126/science.abn8897
	S	S	Regkirona (regdanvimab)	Pseduovirus (HIV)	Wild type (0.2)	13	61	DOI: 10.1016/j.bbrc.2021.09.023
Delta (B.1.617.2)	S	S	Ronapreve (casirivimab / imdevimab)	Virus isolate	Wild type (3.5)	1.9	0.5	DOI: 10.1056/NEJMc2119407
	S	S	Ronapreve (casirivimab / imdevimab)	Pseudovirus (lentivirus)	Wild type (23)	93	4.1	DOI: 10.1101/2022.07.29.502029
	S	S	Ronapreve (casirivimab / imdevimab)	Pseudovirus (HIV)	Wild type (8.1)	20	2.5	DOI: 10.1016/j.isci.2021.103341
	S	S	Ronapreve (casirivimab / imdevimab)	Pseudovirus (HIV)	Wild type (4.4)	9.8	2.2	DOI: 10.3389/fimmu.2022.797589
	S	S	Ronapreve (casirivimab / imdevimab)	Virus isolate	Wild type (30)	20	0.7	https://doi.org/10.1038/s41467-022-28766-y
	S	S	Ronapreve (casirivimab / imdevimab)	Pseudovirus (lentivirus)	Wild type (3.3)	9.1	2.8	DOI: 10.3390/v14061334
	S	S	Ronapreve (casirivimab / imdevimab)	Pseudovirus (MLV)	Wild type (6)	12	2	DOI: 10.1073/pnas.2205784119
	S	S	Xevudy (sotrovimab)	Virus isolate	Wild type (27)	111	4.1	DOI: 10.1056/NEJMc2119407

	S	S	Xevudy (sotrovimab)	Virus isolate	Wild type (46)	52	1.1	DOI: 10.1038/s41598-022-16964-z
	S	S	Xevudy (sotrovimab)	Pseudovirus (lentivirus)	Wild type (564)	1.044	1.9	DOI: 10.1101/2022.07.29.502029
	S	S	Xevudy (sotrovimab)	Pseudovirus (VSV)	Wild type (21)	16	0.8	DOI: 10.1016/j.immuni.2022.06.005
	S	S	Xevudy (sotrovimab)	Virus isolate	Wild type (39)	21	0.5	DOI: 10.1126/scitranslmed.abn6859
	S	S	Xevudy (sotrovimab)	Virus isolate	Wild type (159)	87	0.5	DOI: 10.1101/2022.02.27.482147
	S	S	Xevudy (sotrovimab)	Pseudovirus (HIV)	Wild type (1.964)	219	0.1	DOI: 10.1038/s41591-021-01676-0
	S	S	Xevudy (sotrovimab)	Virus isolate	Wild type (40)	113	2.8	DOI: 10.1016/j.cell.2021.12.046
	S	S	Xevudy (sotrovimab)	Pseudovirus (VSV)	Wild type (11)	32	2.9	DOI: 10.1126/scitranslmed.abn6859
	S	S	Xevudy (sotrovimab)	Pseudovirus (VSV)	Wild type (68)	212	3.1	DOI: 10.1038/s41586-022-04466-x
	S	S	Xevudy (sotrovimab)	Pseudovirus (MLV)	Wild type (72)	162	2.3	DOI: 10.1073/pnas.2205784119
	S	S	Xevudy (sotrovimab)	Virus isolate	Wild type	N/A	1	Xevudy, INN-sotrovimab (europa.eu)
	S	S	Evusheld (tixagevimab / cilgavimab)	Virus isolate	Wild type (3.4)	5.5	1.6	DOI: 10.1056/NEJMc2119407
	S	S	Evusheld (tixagevimab / cilgavimab)	Virus isolate	Wild type (20)	25	1.2	DOI: 10.1038/s41598-022-16964-z
	S	S	Evusheld (tixagevimab / cilgavimab)	Pseudovirus (lentivirus)	Wild type (15)	16	1.1	DOI: 10.1101/2022.07.29.502029
	S	S	Evusheld (tixagevimab / cilgavimab)	Virus isolate	Wild type (9)	5	0.6	DOI: 10.1016/j.cell.2021.12.046
	S	S	Evusheld (tixagevimab / cilgavimab)	Virus isolate	Wild type (8)	3	0.4	Evusheld, INN- tixagevimab, cilgavimab (europa.eu)
	S	S	Evusheld (tixagevimab / cilgavimab)	Virus isolate	Wild type	N/A	1	https://www.fda.gov/media/154701/download
	S	S	Evusheld (tixagevimab / cilgavimab)	Pseudovirus (lentivirus)	Wild type (2.4)	7.5	3.1	DOI: 10.3390/v14061334
	S	S	Evusheld (tixagevimab / cilgavimab)	Pseudovirus	Wild type (1.9)	1.9	1	Evusheld, INN- tixagevimab, cilgavimab (europa.eu)
	S	S	Regkirona (regdanvimab)	Pseudovirus (lentivirus)	Wild type (19)	110	5.9	DOI: 10.1101/2022.07.29.502029

	S	S	Regkirona (regdanvimab)	Pseudovirus (VSV)	Wild type (2.2)	16	7.4	DOI: 10.1038/s41586-021-03944-y
	S	S	Regkirona (regdanvimab)	Virus isolate	Wild type	N/A	183	Regkirona, INN-regdanvimab (europa.eu)
	S	S	Regkirona (regdanvimab)	Pseudovirus (lentivirus)	Wild type (1.5)	15	9.8	DOI: 10.1126/science.abn8897
Omicron BA.1	S	S	Ronapreve (casirivimab / imdevimab)	Pseudovirus (lentivirus)	Wild type (0.8)	>5000	>1000	https://doi.org/10.1016/j.ebiom.2022.103944
	S	S	Ronapreve (casirivimab / imdevimab)	Pseudovirus (VSV)	Wild type (7.6)	2372	312	https://doi.org/10.1016/j.chom.2022.04.017
	S	S	Ronapreve (casirivimab / imdevimab)	Pseudovirus (lentivirus)	Wild type (6.6)	1469	223	https://doi.org/10.1016/S1473-3099(22)00422-4
	S	S	Ronapreve (casirivimab / imdevimab)	Pseudovirus (VSV)	Wild type (1)	2951	>1000	https://doi.org/10.1038/s41586-022-05053-w
	S	S	Ronapreve (casirivimab / imdevimab)	Pseudovirus (HIV)	Wild type (3.1)	>10000	>1000	10.1016/j.cell.2022.04.035
	S	S	Ronapreve (casirivimab / imdevimab)	Virus isolate	Wild type (3.5)	>10000	>1000	https://doi.org/10.1056/NEJMc2119407
	S	S	Ronapreve (casirivimab / imdevimab)	Virus isolate	Delta (4.8)	>10000	>1000	https://doi.org/10.1038/s41586-022-04474-x
	S	S	Ronapreve (casirivimab / imdevimab)	Virus isolate	Delta (8)	>20000	>1000	https://doi.org/10.1101/2022.03.18.484873
	S	S	Ronapreve (casirivimab / imdevimab)	Pseudovirus (VSV)	Wild type (1)	>10000	>1000	https://doi.org/10.1038/s41586-021-04388-0
	S	S	Ronapreve (casirivimab / imdevimab)	Virus isolate	Wild type (0.1)	>12.307	>1000	https://doi.org/10.1093/cid/ciac143
	S	S	Xevudy (sotrovimab)	Pseudovirus (lentivirus)	Wild type (123)	2334	19	https://doi.org/10.1016/j.ebiom.2022.103944
	S	S	Xevudy (sotrovimab)	Pseudovirus (lentivirus)	Wild type (36)	281	7.8	https://doi.org/10.1126/science.abn8897
	S	S	Xevudy (sotrovimab)	Pseudovirus (VSV)	Wild type (78)	356	4.5	https://doi.org/10.1101/2022.02.07.479349
	S	S	Xevudy (sotrovimab)	Pseudovirus (VSV)	Wild type (12)	49	4.1	https://doi.org/10.1126/scitranslmed.abn6859
	S	S	Xevudy (sotrovimab)	Pseudovirus (lentivirus)	Wild type (3)	10	3.3	https://doi.org/10.1101/2022.05.25.493397

	S	S	Xevudy (sotrovimab)	Pseudovirus (HIV)	Wild type (12)	57	4.8	https://doi.org/10.1016/j.cell.2022.04.035
	S	S	Xevudy (sotrovimab)	Pseudovirus (lentivirus)	Wild type (140)	1480	11	https://doi.org/10.3390/v14061334
	S	S	Xevudy (sotrovimab)	Pseudovirus (HIV)	Wild type (31)	192	62	https://doi.org/10.1101/2022.01.30.478305
	S	S	Xevudy (sotrovimab)	Pseudovirus	Wild type	N/A	1	Xevudy, INN-sotrovimab (europa.eu)
	S	S	Xevudy (sotrovimab)	Pseudovirus	Wild type	N/A	1	https://www.fda.gov/media/149534/download
	S	S	Xevudy (sotrovimab)	Virus isolate	Wild type (89)	316	6.9	https://doi.org/10.1038/s41598-022-16964-z
	S	S	Xevudy (sotrovimab)	Virus isolate	Wild type (27)	373	14	https://doi.org/10.1056/NEJMc2119407
	S	S	Xevudy (sotrovimab)	Virus isolate	Wild type (185)	452	2.4	https://doi.org/10.1038/s41467-022-31615-7
	S	S	Xevudy (sotrovimab)	Virus isolate	Wild type (40)	256	6.4	https://doi.org/10.1016/j.cell.2021.12.046
	S	S	Xevudy (sotrovimab)	Pseudovirus (HIV)	Wild type (100)	200	2	https://doi.org/10.1016/S1473-3099(22)00129-3
	S	S	Evusheld (tixagevimab / cilgavimab)	Pseudovirus (lentivirus)	Wild type (14)	1100	81	10.1016/j.ebiom.2022.103944
	S	S	Evusheld (tixagevimab / cilgavimab)	Pseudovirus (VSV)	Wild type (5.9)	170	29	0.1016/j.chom.2022.04.017
	S	S	Evusheld (tixagevimab / cilgavimab)	Pseudovirus (lentivirus)	Wild type (1.3)	256	197	https://doi.org/10.1126/scitranslmed.abn8543
	S	S	Evusheld (tixagevimab / cilgavimab)	Pseudovirus (VSV)	Wild type (2)	154	77	https://doi.org/10.1038/s41586-022-05053-w
	S	S	Evusheld (tixagevimab / cilgavimab)	Pseudovirus (lentivirus)	Wild type (2.4)	862	359	https://doi.org/10.3390/v14061334
	S	S	Evusheld (tixagevimab / cilgavimab)	Pseudovirus (lentivirus)	Wild type (1)	232	232	https://doi.org/10.1016/j.cell.2022.06.005
	S	S	Evusheld (tixagevimab / cilgavimab)	Pseudovirus	Wild type (1.9)	51	27	Evusheld, INN- tixagevimab, cilgavimab (europa.eu)
	S	S	Evusheld (tixagevimab / cilgavimab)	Pseudovirus (MLV)	Wild type (5)	1313	263	https://doi.org/10.1073/pnas.2205784119
	S	S	Evusheld (tixagevima	Pseudovirus	Wild type	N/A	158	https://www.fda.gov/media/154701/download

			b / cilgavimab)					
	S	S	Evusheld (tixagevimab / cilgavimab)	Virus isolate	Delta (2.6)	715	275	https://doi.org/10.1038/s41591-022-01792-5
	S	S	Evusheld (tixagevimab / cilgavimab)	Virus isolate	Wild type (3.4)	256	75	https://doi.org/10.1056/NEJMc2119407
	S	S	Evusheld (tixagevimab / cilgavimab)	Virus isolate	Wild type (6.5)	167	26	https://doi.org/10.1038/s41467-022-31615-7
	S	S	Evusheld (tixagevimab / cilgavimab)	Virus isolate	Wild type (9)	273	30	https://doi.org/10.1016/j.cell.2021.12.046
	S	S	Evusheld (tixagevimab / cilgavimab)	Virus isolate	Wild type (8)	147	18	Evusheld, INN- tixagevimab, cilgavimab (europa.eu)
	S	S	Evusheld (tixagevimab / cilgavimab)	Pseudovirus (VSV)	Wild type (0.5)	50	101	https://doi.org/10.1038/s41586-021-04388-0
	S	S	Regkirona (regdanvimab)	Pseudovirus (VSV)	Wild type (1.5)	>10,000	>1000	https://doi.org/10.1016/j.celrep.2022.110812
	S	S	Regkirona (regdanvimab)	Psedudovirus (lentivirus)	Wild type (4.9)	>10,000	>1000	https://doi.org/10.1016/j.chom.2022.04.017
	S	S	Regkirona (regdanvimab)	Pseudovirus (HIV)	Wild type (5)	>10,000	>1000	https://doi.org/10.1016/j.chom.2022.07.002
	S	S	Regkirona (regdanvimab)	Virus isolate	Wild type (13)	>1,000	>77	https://doi.org/10.1038/s41598-022-08559-5
	S	S	Regkirona (regdanvimab)	Virus isolate	Wild type (2.4)	>10,000	>1000	https://doi.org/10.1038/s41591-021-01678-y
Omicron BA.2	S	S	Ronapreve (casirivimab / imdevimab)	Pseudovirus (VSV)	Wild type (7.6)	486	64	https://doi.org/10.1016/j.chom.2022.04.017
	S	S	Ronapreve (casirivimab / imdevimab)	Pseudovirus (VSV)	Wild type (5)	821	164	https://doi.org/10.1101/2022.07.18.500332
	S	S	Ronapreve (casirivimab / imdevimab)	Pseudovirus (VSV)	Wild type (6.7)	2303	344	https://doi.org/10.1038/s41586-022-04594-4
	S	S	Ronapreve (casirivimab / imdevimab)	Pseudovirus (HIV)	Wild type (3.1)	>10,000	>1000	https://doi.org/10.1016/j.cell.2022.04.035
	S	S	Ronapreve (casirivimab / imdevimab)	Virus isolate	Delta (8)	980	123	https://doi.org/10.1101/2022.03.18.484873
	S	S	Ronapreve (casirivimab / imdevimab)	Virus isolate	Wild type (3.5)	223	63	https://doi.org/10.1056/NEJMc2201933
	S	S	Ronapreve (casirivimab / imdevimab)	Virus isolate	Wild type (2.2)	968	434	https://doi.org/10.1056/NEJMc2209952

	S	S	Xevudy (sotrovimab)	Pseudovirus	Wild type (28)	2118	76	https://doi.org/10.1016/S1473-3099(22)00580-1
	S	S	Xevudy (sotrovimab)	Pseudovirus (VSV)	Wild type (198)	7,032	36	https://doi.org/10.1016/j.chom.2022.04.017
	S	S	Xevudy (sotrovimab)	Pseudovirus (VSV)	Wild type (74)	918	12	https://doi.org/10.1101/2022.07.18.500332
	S	S	Xevudy (sotrovimab)	Pseudovirus (lentivirus)	Wild type (564)	14,907	26	https://doi.org/10.1101/2022.07.29.502029
	S	S	Xevudy (sotrovimab)	Pseudovirus (VSV)	Wild type (25)	558	22	https://doi.org/10.1016/j.chom.2022.09.002
	S	S	Xevudy (sotrovimab)	Pseudovirus (lentivirus)	Wild type (3)	400	133	https://doi.org/10.1016/j.cell.2022.04.035
	S	S	Xevudy (sotrovimab)	Pseudovirus (VSV)	Wild type (9)	160	18	https://doi.org/10.1016/j.chom.2022.05.001
	S	S	Xevudy (sotrovimab)	Pseudovirus	Wild type	N/A	16	Xevudy, INN-sotrovimab (europa.eu)
	S	S	Xevudy (sotrovimab)	Virus isolate	Delta (147)	2200	15	https://doi.org/10.1101/2022.03.18.484873
	S	S	Xevudy (sotrovimab)	Virus isolate	Wild type	N/A	35	https://www.ema.europa.eu/en/documents/product-information/xevudy-epar-product-information_en.pdf
	S	S	Xevudy (sotrovimab)	Virus isolate	Wild type (33)	>50,000	>1000	10.1056/NEJMc2209952
	S	S	Xevudy (sotrovimab)	Virus isolate	Wild type (185)	5,885	32	https://doi.org/10.1038/s41467-022-31615-7
	S	S	Evusheld (tixagevimab / cilgavimab)	Pseudovirus (VSV)	Wild type (7.6)	71	9.3	https://doi.org/10.1016/S1473-3099(22)00422-4
	S	S	Evusheld (tixagevimab / cilgavimab)	Pseudovirus (VSV)	Wild type (2.1)	8.2	3.9	https://doi.org/10.1101/2022.07.18.500332
	S	S	Evusheld (tixagevimab / cilgavimab)	Pseudovirus (lentivirus)	Wild type (15)	49	3.3	https://doi.org/10.1101/2022.07.29.502029
	S	S	Evusheld (tixagevimab / cilgavimab)	Pseudovirus (VSV)	Wild type (2)	22	11	https://doi.org/10.1016/j.chom.2022.09.002
	S	S	Evusheld (tixagevimab / cilgavimab)	Pseudovirus	Wild type (4.7)	42	8.9	https://doi.org/10.1101/2022.07.14.500041
	S	S	Evusheld (tixagevimab / cilgavimab)	Pseudovirus	Wild type (1.9)	9.8	5.2	Evusheld, INN- tixagevimab, cilgavimab (europa.eu)

	S	S	Evusheld (tixagevimab / cilgavimab)	Virus isolate	Wild type (8)	35	4.4	Evusheld, INN- tixagevimab, cilgavimab (europa.eu)
	S	S	Evusheld (tixagevimab / cilgavimab)	Virus isolate	Wild type (20)	37	19	https://doi.org/10.1038/s41598-022-16964-z
	S	S	Evusheld (tixagevimab / cilgavimab)	Virus isolate	Delta (2.6)	23	8.8	https://doi.org/10.1038/s41591-022-01792-5
	S	S	Evusheld (tixagevimab / cilgavimab)	Virus isolate	Wild type (6.5)	43	6.7	10.1056/NEJMc2209952
	S	S	Regkirona (regdanvimab)	Pseudovirus (VSV)	Wild type (4.9)	>10,000	>1000	https://doi.org/10.1016/j.chom.2022.04.017
	S	S	Regkirona (regdanvimab)	Pseudovirus (lentivirus)	Wild type (19)	>67,000	>1000	https://doi.org/10.1101/2022.07.29.502029
	S	S	Regkirona (regdanvimab)	Pseudovirus	Wild type (1)	>4,025	>1000	https://doi.org/10.1101/2022.07.14.500041
	S	S	Regkirona (regdanvimab)	Pseudovirus (VSV)	Wild type (1)	>10,000	>1000	https://doi.org/10.1016/j.chom.2022.05.001
	S	S	Regkirona (regdanvimab)	Virus isolate	Delta (23)	>9,000	>391	https://doi.org/10.1038/s41591-022-01792-5
BA.4	S	S	Ronapreve (casirivimab / imdevimab)	Virus isolate	Wild type (3.4)	459	135	https://doi.org/10.1056/NEJMc2207519
	S	S	Ronapreve (casirivimab / imdevimab)	Virus isolate	Delta (2)	660	330	DOI: 10.1016/j.xcrm.2022.100850
	S	S	Xevudy (sotrovimab)	Virus isolate	Wild type (95)	>50,000	>526	https://doi.org/10.1056/NEJMc2207519
	S	S	Xevudy (sotrovimab)	Pseudovirus (VSV)	Wild Type (69)	1,885	27	DOI: 10.1126/science.adc9127
	S	S	Xevudy (sotrovimab)	Virus isolate	Delta (64)	996	16	DOI: 10.1016/j.xcrm.2022.100850
	S	S	Evusheld (tixagevimab / cilgavimab)	Virus isolate	Wild type (6.3)	38	6	https://doi.org/10.1056/NEJMc2207519
	S	S	Evusheld (tixagevimab / cilgavimab)	Virus isolate	Delta (2.5)	26	10	DOI: 10.1016/j.xcrm.2022.100850
Omicron BA.5	S	S	Ronapreve (casirivimab / imdevimab)	Pseudovirus	Omicron BA.2 (821)	709	0.9	https://doi.org/10.1101/2022.09.15.507787
	S	S	Ronapreve (casirivimab / imdevimab)	Pseudovirus (VSV)	Wild type (6.6)	966	146	https://doi.org/10.1016/S1473-3099(22)00422-4

	S	S	Ronapreve (casirivimab / imdevimab)	Pseudovirus (lentivirus)	Wild type (23)	9,795	432	https://doi.org/10.1101/2022.07.29.502029
	S	S	Ronapreve (casirivimab / imdevimab)	Pseudovirus (VSV)	Wild type (1)	1,998	>1000	https://doi.org/10.1038/s41586-022-05053-w
	S	S	Ronapreve (casirivimab / imdevimab)	Pseudovirus (VSV)	Wild type (3)	2,779	926	https://doi.org/10.1016/j.chom.2022.09.002
	S	S	Ronapreve (casirivimab / imdevimab)	Pseudovirus	Wild type (3.9)	>5,000	>1000	https://doi.org/10.1101/2022.07.14.500041
	S	S	Ronapreve (casirivimab / imdevimab)	Virus isolate	Omicron BA.4 (2000)	2,800	1.4	https://doi.org/10.1101/2022.07.28.501852
	S	S	Xevudy (sotrovimab)	Virus isolate	Wild type 46)	858	19	https://doi.org/10.1038/s41598-022-16964-z
	S	S	Xevudy (sotrovimab)	Pseudovirus	Omicron BA.2 (852)	>1,055	1	https://doi.org/10.1101/2022.09.15.507787
	S	S	Xevudy (sotrovimab)	Pseudovirus (lentivirus)	Wild type (564)	10,232	18	https://doi.org/10.1101/2022.07.29.502029
	S	S	Xevudy (sotrovimab)	Pseudovirus (VSV)	Wild type (14)	1,120	80	https://doi.org/10.1038/s41586-022-05053-w
	S	S	Xevudy (sotrovimab)	Pseudovirus (lentivirus)	Wild type (3)	142	47	https://doi.org/10.1101/2022.05.25.493397
	S	S	Xevudy (sotrovimab)	Pseudovirus (HIV)	Wild type (94)	1,261	13	https://doi.org/10.1016/S1473-3099(22)00365-6
	S	S	Xevudy (sotrovimab)	Pseudovirus (lentivirus)	Wild type (130)	1,041	8	https://doi.org/10.1016/j.cell.2022.06.005
	S	S	Xevudy (sotrovimab)	Pseudovirus	Wild type	N/A	21	https://www.ema.europa.eu/en/documents/product-information/evusheld-epar-product-information_en.pdf
	S	S	Xevudy (sotrovimab)	Virus isolate	Wild type (33)	6,240	190	https://doi.org/10.1056/NEJMc2209952
	S	S	Evusheld (tixagevimab / cilgavimab)	Virus isolate	Wild type (20)	57	2.8	https://doi.org/10.1038/s41598-022-16964-z
	S	S	Evusheld (tixagevimab / cilgavimab)	Pseudovirus	Omicron BA.2 (8.2)	40	4.9	https://doi.org/10.1101/2022.09.15.507787
	S	S	Evusheld (tixagevimab / cilgavimab)	Pseudovirus (lentivirus)	Wild type (15)	314	21	https://doi.org/10.1101/2022.07.29.502029
	S	S	Evusheld (tixagevima	Pseudovirus (VSV)	Wild type (2)	49	25	https://doi.org/10.1038/s41586-022-05053-w

			b / cilgavimab)					
	S	S	Evusheld (tixagevimab / cilgavimab)	Pseudovirus (HIV)	Wild type (4.1)	609	149	https://doi.org/10.1016/S1473-3099(22)00365-6
	S	S	Evusheld (tixagevimab / cilgavimab)	Pseudovirus (lentivirus)	Wild type (1)	65	65	https://doi.org/10.1016/j.cell.2022.06.005
	S	S	Evusheld (tixagevimab / cilgavimab)	Virus isolate	Omicron BA.4 (249)	281	1.1	https://doi.org/10.1101/2022.07.28.501852
	S	S	Evusheld (tixagevimab / cilgavimab)	Virus isolate	Wild type (6.5)	124	19	https://doi.org/10.1056/NEJMc2209952
	S	S	Regkirona (regdanvimab)	Pseudovirus	Wild type (5)	>10,000	>1000	https://doi.org/10.1016/S1473-3099(22)00580-1
	S	S	Regkirona (regdanvimab)	Pseudovirus (VSV)	Wild type (2.9)	>5,000	>1000	https://doi.org/10.1016/S1473-3099(22)00422-4
	S	S	Regkirona (regdanvimab)	Pseudovirus (lentivirus)	Wild type (19)	>67,000	>1000	https://doi.org/10.1101/2022.07.29.502029
	S	S	Regkirona (regdanvimab)	Pseudovirus	Wild type (1)	>4,025	>1000	https://doi.org/10.1101/2022.07.14.500041
Omicron BA.2.75	S	S	Ronapreve (casirivimab / imdevimab)	Pseudovirus	Omicron BA. 2 (821)	410	0.5	https://doi.org/10.1101/2022.09.15.507787
	S	S	Ronapreve (casirivimab / imdevimab)	Pseudovirus (VSV)	Wild type (3)	3,504	>1000	https://doi.org/10.1016/j.chom.2022.09.002
	S	S	Ronapreve (casirivimab / imdevimab)	Pseudovirus	Wild type (3.9)	>5,000	>1000	https://doi.org/10.1101/2022.07.14.500041
	S	S	Ronapreve (casirivimab / imdevimab)	Virus isolate	Wild type (2.2)	1,812	823	https://doi.org/10.1056/NEJMc2209952
	S	S	Xevudy (sotrovimab)	Pseudovirus	Omicron BA.2 (852)	672	0.8	https://doi.org/10.1101/2022.09.15.507787
	S	S	Xevudy (sotrovimab)	Pseudovirus (lentivirus)	Wild type (78)	>1,000	>13	https://doi.org/10.1101/2022.07.19.500716
	S	S	Xevudy (sotrovimab)	Pseudovirus (VSV)	Wild type (25)	467	19	https://doi.org/10.1016/j.chom.2022.09.002
	S	S	Xevudy (sotrovimab)	Virus isolate	Wild type (33)	28,536	870	https://doi.org/10.1056/NEJMc2209952
	S	S	Evusheld (tixagevimab / cilgavimab)	Pseudovirus	Omicron BA. 2 (8.2)	121	15	https://doi.org/10.1101/2022.09.15.507787
	S	S	Evusheld (tixagevimab / cilgavimab)	Pseudovirus (VSV)	Wild type (2)	49	25	https://doi.org/10.1016/j.chom.2022.09.002

	S	S	Evusheld (tixagevimab / cilgavimab)	Pseudovirus	Wild type (4.7)	113	24	https://doi.org/10.1101/2022.07.14.500041
	S	S	Evusheld (tixagevimab / cilgavimab)	Virus isolate	Wild type (6.5)	34	5.3	https://doi.org/10.1056/NEJMc2209952
	S	S	Regkirona (regdanvimab)	Pseudovirus	Wild type (5)	40	8	https://doi.org/10.1016/S1473-3099(22)00580-1
	S	S	Regkirona (regdanvimab)	Pseudovirus	Wild type (1)	42	42	https://doi.org/10.1101/2022.07.14.500041
	S	S	Ronapreve (casirivimab / imdevimab)	Pseudovirus	Omicron BA.2 (821)	>1,000	1	https://doi.org/10.1101/2022.09.15.507787
	S	S	Ronapreve (casirivimab / imdevimab)	Pseudovirus	Wild type (5)	1000	200	DOI: 10.1038/s41586-022-05644-7
	S	S	Xevudy (sotrovimab)	Pseudovirus	Omicron BA.2 (852)	>1,709	2	https://doi.org/10.1101/2022.09.15.507787
	S	S	Xevudy (sotrovimab)	Pseudovirus (VSV)	Wild type (23)	6,000	261	DOI: 10.1016/j.cell.2022.12.018
	S	S	Xevudy (sotrovimab)	Virus isolate	Delta (99)	688	7	DOI: 10.1016/j.isci.2023.106413
	S	S	Xevudy (sotrovimab)	Pseudovirus	Wild type (32)	1,535	48	DOI: 10.1101/2023.04.08.536123
	S	S	Xevudy (sotrovimab)	Pseudovirus (VSV)	Wild type (18)	1,247	22	DOI: 10.1101/2023.01.17.523798
	S	S	Xevudy (sotrovimab)	Virus isolate	Delta (99)	688	7	DOI: 10.1101/2022.12.22.521201
	S	S	Evusheld (tixagevimab / cilgavimab)	Pseudovirus	Omicron BA.2 (8.2)	>1,000	>122	https://doi.org/10.1101/2022.09.15.507787
	S	S	Evusheld (tixagevimab / cilgavimab)	Virus isolate	Delta (13)	>5000	400	DOI: 10.1016/j.isci.2023.106413
	S	S	Evusheld (tixagevimab / cilgavimab)	Pseudovirus	Wild type	NA	1000	DOI: 10.1101/2023.02.07.527406
	S	S	Evusheld (tixagevimab / cilgavimab)	Pseudovirus	Wild type (2.1)	>1,000	>476	https://doi.org/10.1101/2022.09.15.507787
Omicron BQ.1	S	S	Evusheld (tixagevimab / cilgavimab)	Pseudovirus	Wild type	N/A	>2000	https://www.fda.gov/media/154701/download
	S	S	Ronapreve (casirivimab / imdevimab)	Pseudovirus	Omicron BA.2 (821)	>1,000	1	https://doi.org/10.1101/2022.09.15.507787
Omicron BQ.1.1	S	S	Ronapreve (casirivimab / imdevimab)	Pseudovirus	Wild type (5)	1000	200	DOI: 10.1038/s41586-022-05644-7

	S	S	Ronapreve (casirivimab / imdevimab)	Pseudovirus	Wild type (9)	50000	1000	DOI: 10.1016/S1473-3099(22)00733-2
	S	S	Xevudy (sotrovimab)	Pseudovirus	Omicron BA.2 (852)	5,581	7	https://doi.org/10.1101/2022.09.15.507787
	S	S	Xevudy (sotrovimab)	Pseudovirus	Wild type (74)	5,581	75	DOI: 10.1038/s41586-022-05644-7
	S	S	Xevudy (sotrovimab)	Virus isolate	Wild type (328)	100,000	300	DOI: 10.1016/j.ebiom.2023.104545
	S	S	Xevudy (sotrovimab)	Virus isolate	Omicron BA.1 (292)	4,440	15	DOI: 10.1016/j.ccell.2023.04.005
	S	S	Xevudy (sotrovimab)	Virus isolate	Delta (99)	1,238	13	DOI: 10.1016/j.isci.2023.106413
	S	S	Xevudy (sotrovimab)	Virus isolate	Wild type (65)	7,640	118	DOI: 10.1038/s41467-023-36561-6
	S	S	Evusheld (tixagevimab / cilgavimab)	Pseudovirus	Omicron BA.2 (8.2)	>1,000	>122	https://doi.org/10.1101/2022.09.15.507787
	S	S	Evusheld (tixagevimab / cilgavimab)	Pseudovirus	Wild type (2.1)	>1,000	>476	https://doi.org/10.1101/2022.09.15.507787
	S	S	Evusheld (tixagevimab / cilgavimab)	Pseudovirus	Wild type	N/A	>2000	https://www.fda.gov/media/154701/download
	S	S	Evusheld (tixagevimab / cilgavimab)	Virus isolate	Omicron BA.1 (291)	250000	859	DOI: 10.1016/j.ccell.2023.04.005
	S	S	Evusheld (tixagevimab / cilgavimab)	Virus isolate	Delta (13)	5000	400	DOI: 10.1016/j.isci.2023.106413
	S	S	Ronapreve (casirivimab / imdevimab)	Pseudovirus	Omicron BA.2 (821)	>1000	>1	DOI: 10.1038/s41586-022-05644-7
	S	S	Ronapreve (casirivimab / imdevimab)	Pseudovirus	Wild type (5)	>1000	>200	DOI: 10.1038/s41586-022-05644-7
	S	S	Xevudy (sotrovimab)	Virus isolate	Delta (99)	807	8.2	https://doi.org/10.1016/j.isci.2023.106413
	S	S	Xevudy (sotrovimab)	Virus isolate	Omicron BA.1 (292)	269	0.9	doi:10.1016/j.ccell.2023.04.005
	S	S	Xevudy (sotrovimab)	Pseudovirus	Wild type (74)	963	13	DOI: 10.1038/s41586-022-05644-7
	S	S	Xevudy (sotrovimab)	Pseudovirus	Omicron BA.2 (852)	963	1.1	DOI: 10.1038/s41586-022-05644-7
Omicron XBB	S	S	Xevudy (sotrovimab)	Pseudovirus (VSV)	Omicron BA.2 (21)	171	8.1	DOI: 10.1016/j.xcrm.2023.100991

	S	S	Evusheld (tixagevimab / cilgavimab)	Pseudovirus	Omicron BA.2 (8.2)	>1000	>122	DOI: 10.1038/s41586-022-05644-7
	S	S	Evusheld (tixagevimab / cilgavimab)	Pseudovirus	Wild type (2.1)	>1000	>476	DOI: 10.1038/s41586-022-05644-7
Omicron XBB.1.5	S	S	Ronapreve (casirivimab / imdevimab)	Virus isolate	Omicron BA.2 (6.299)	50 000	8	DOI: 10.1016/S1473-3099(23)00070-1
	S	S	Ronapreve (casirivimab / imdevimab)	Virus isolate	Wild type (56)	50 000	887	DOI: 10.1016/S1473-3099(23)00070-1
	S	S	Ronapreve (casirivimab / imdevimab)	Pesudovirus	Wild type (7.9)	4860	615	DOI: 10.1101/2023.04.06.535883
	S	S	Xevudy (sotrovimab)	Virus isolate	Wild type (1.584)	50000	32	DOI: 10.1016/S1473-3099(23)00070-1
	S	S	Xevudy (sotrovimab)	Virus isolate	Omicron BA.1 (292)	197	0.7	DOI: 10.1016/j.ccell.2023.04.005
	S	S	Xevudy (sotrovimab)	Pseudovirus (VSV)	Wild type (58)	436	7.5	DOI: 10.1101/2023.01.17.523798
	S	S	Xevudy (sotrovimab)	Pseudovirus	Omciron XBB.1 (896)	915	1	DOI: 10.1101/2023.01.03.522427
	S	S	Xevudy (sotrovimab)	Pseudovirus	Wil type (32)	575	18	DOI: 10.1101/2023.04.08.536123
	S	S	Xevudy (sotrovimab)	Pseduovirus (lentivirus)	Wild type	740	5	DOI: 10.1101/2023.04.06.535883
	S	S	Evusheld (tixagevimab / cilgavimab)	Virus isolate	Wild type (58)	50000	867	DOI: 10.1016/S1473-3099(23)00070-1
	S	S	Evusheld (tixagevimab / cilgavimab)	Virus isolate	Omicron BA.1 (291)	250000	859	DOI: 10.1016/j.ccell.2023.04.005
	S	S	Evusheld (tixagevimab / cilgavimab)	Pseudovirus (VSV)	Wild type (NA)	NA	1000	DOI: 10.1101/2023.02.07.527406
	S	S	Evusheld (tixagevimab / cilgavimab)	Pseudovirus (lentivirus)	Wild type (4.3)	2100	488	DOI: 10.1101/2023.04.06.535883
Omicron BA.2.86	S	S	Sotrovimab	Pseudovirus	Omicron BA.2 (559)	10000	18	DOI: 10.1101/2023.09.02.556033
	S	S	Sotrovimab	Pseudovirus	Wild type (200)	26042	130	DOI: 10.1101/2023.09.07.556636

	S	S	Sotrovimab	Pseudovirus	Omicron XBB 1.5 (900)	1890	2.1	DOI: 10.1101/2023.09.01.555815
	S	S	Evusheld	Pseudovirus	Wild type	1.000.000	185	DOI: 10.1101/2023.09.07.556636
	S	S	Evusheld	Pseudovirus	XBB.1.5 (4)	10	1000	https://doi.org/10.1016/S1473-3099(23)00573-X
